# Guidance for the treatment of deep vein thrombosis and pulmonary embolism

**DOI:** 10.1007/s11239-015-1317-0

**Published:** 2016-01-16

**Authors:** Michael B. Streiff, Giancarlo Agnelli, Jean M. Connors, Mark Crowther, Sabine Eichinger, Renato Lopes, Robert D. McBane, Stephan Moll, Jack Ansell

**Affiliations:** Division of Hematology, Department of Medicine and Pathology, The Johns Hopkins University School of Medicine, Baltimore, MD USA; Stroke Unit, Department of Internal Medicine, University of Perugia, Perugia, Italy; Hematology Division, Brigham and Women’s Hospital, Dana Farber Cancer Institute, Harvard Medical School, Boston, MA USA; Departments of Medicine and Pathology and Molecular Medicine, McMaster University, Hamilton, Canada; Department of Medicine, Medical University of Vienna, Vienna, Austria; Division of Cardiology, Department of Medicine, Duke University Medical Center, Durham, NC USA; Cardiovascular Division, Department of Medicine, Mayo Clinic, Rochester, MN USA; Department of Medicine, University of North Carolina School of Medicine, Chapel Hill, NC USA; Department of Medicine, Hofstra North Shore/LIJ School of Medicine, Hempstead, NY USA

**Keywords:** Anticoagulant therapy, Venous thromboembolism, Deep vein thrombosis, Pulmonary embolism, NOACs, DOACs

## Abstract

This guidance document focuses on the diagnosis and treatment of venous thromboembolism (VTE). Efficient, cost effective diagnosis of VTE is facilitated by combining medical history and physical examination with pre-test probability models, D dimer testing and selective use of confirmatory imaging. Clinical prediction rules, biomarkers and imaging can be used to tailor therapy to disease severity. Anticoagulation options for acute VTE include unfractionated heparin, low molecular weight heparin, fondaparinux and the direct oral anticoagulants (DOACs). DOACs are as effective as conventional therapy with LMWH and vitamin K antagonists. Thrombolytic therapy is reserved for massive pulmonary embolism (PE) or extensive deep vein thrombosis (DVT). Inferior vena cava filters are reserved for patients with acute VTE and contraindications to anticoagulation. Retrievable filters are strongly preferred. The possibility of thoracic outlet syndrome and May-Thurner syndrome should be considered in patients with subclavian/axillary and left common iliac vein DVT, respectively in absence of identifiable triggers. The optimal duration of therapy is dictated by the presence of modifiable thrombotic risk factors. Long term anticoagulation should be considered in patients with unprovoked VTE as well as persistent prothrombotic risk factors such as cancer. Short-term therapy is sufficient for most patients with VTE associated with transient situational triggers such as major surgery. Biomarkers such as D dimer and risk assessment models such the Vienna risk prediction model offer the potential to customize VTE therapy for the individual patient. Insufficient data exist to support the integration of bleeding risk models into duration of therapy planning.

## Introduction

Venous thromboembolism (VTE) which consists principally of deep vein thrombosis (DVT) and pulmonary embolism (PE) is a common cause of morbidity and mortality. Consequently, health care providers in all clinical settings will be faced with managing patients with this illness. Numerous evidence-based guidelines are available to assist providers in clinical decision-making. However, there are many clinical scenarios where a paucity of data exist. The purpose of this guidance document is to provide advice to providers on all aspects of the treatment of VTE based upon the best available information including situations where evidence is limited.

**Fig. 1:**
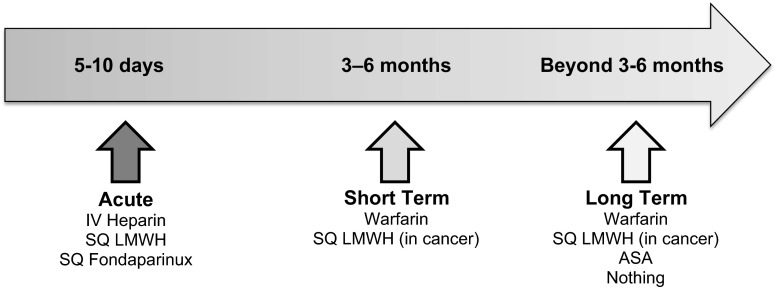
The different phases of treatment and traditional therapies in venous thromboembolism

Many authorities divide the therapy of VTE into various phases of treatment following the initial diagnosis based upon the risk of recurrence. For the purposes of this guidance document, we consider the initial treatment of VTE, the “acute” phase, to encompass the first 5–10 days which corresponds to the time period when patients historically have been treated with parenteral therapy. The next 3–6 months, we consider the “short term” treatment phase of therapy. After 3–6 months, we apply the term “long term” treatment of VTE when the benefit/risk of continued treatment becomes a critical aspect of the decision making process. Figure 1 illustrates this continuum of care.

## Methods

To provide guidance on the management of VTE, the authors developed a list of important management questions to be considered in this document (Table 1). Questions were developed by consensus of all the authors. To answer these questions, a literature search of MEDLINE and EMBASE from January 2004 to August 2014 was conducted. The following search terms were used and combined: anticoagulant treatment, anticoagulant therapy, antithrombotic treatment, heparin, low molecular weight heparin, enoxaparin, nadroparin, dalteparin, certoparin, bemiparin, tinzaparin, parnaparin, reviparin, vitamin K antagonists, warfarin, acenocoumarol, phenprocoumon, thrombolysis, thrombolytic treatment, fibrinolytic agent, fibrinolysis, urokinase, tenecteplase, alteplase, rtPA, tPA; aspirin, ticlopidine, clopidogrel; venous thromboembolism, venous thrombosis, deep venous thrombosis, deep vein thrombosis, superficial venous thrombosis, superficial venous thrombophlebitis; diagnosis. The search strategy was restricted to papers published in English. Detailed information on the results of the literature search is available upon request.

**Table 1:** Guidance questions to be considered

How is the diagnosis of deep vein thrombosis and pulmonary embolism established?
Which patients require hospitalization versus initial outpatient therapy for the management of VTE?
What are the therapeutic options for the acute treatment of venous thromboembolism?
Which patients are candidates for a DOAC?
What is the role of vena cava filters if the patient is not a candidate for anticoagulation?
How is upper extremity VTE treated?
When is ambulation/exercise safe after DVT/PE?
Is the use of graduated compression stockings safe after acute DVT/PE?
What is the recommended duration of therapy for VTE? What is the recommended duration of therapy for a patient with distal DVT? What is the recommended duration of therapy for a patient with a surgically provoked VTE? What is the recommended duration of therapy for a pregnancy or estrogen-associated VTE? What is the recommended duration of therapy for a medical illness-associated VTE? What is the recommended duration of therapy for a travel-associated VTE? What is the recommended duration of therapy for a malignancy-associated VTE? What is the recommended duration of therapy for a patient with unprovoked DVT/PE?
What are the therapeutic options for long term treatment of DVT/PE?
What is the best treatment of patients who have recurrent VTE in spite of anticoagulation?
How can you assess the risk of recurrent VTE and anticoagulant-associated bleeding?

For papers published before 2004, we only considered the most important studies that were likely to influence our responses to the questions. These studies were selected and suggested by the authors of this guidance document.

## Guidance

1. How is the diagnosis of deep vein thrombosis and pulmonary embolism established?

Deep vein thrombosis should be suspected in any patient who presents with unexplained extremity swelling, pain, warmth or erythema. Pain associated with DVT is often described as being a cramp or ache in the calf or thigh. Pulmonary embolism is often heralded by development of dyspnea and pleuritic chest or back pain. Pulmonary embolism can also cause progressive fatigue, dyspnea on exertion, syncope or pre-syncope or sudden death. Since these symptoms can be caused by many diseases, the likelihood of VTE can be estimated by assessing a patient’s thrombosis risk factors (Table 2) [1, 2]. The presence of these disease processes should be elicited in the history when assessing a patient for VTE.

**Table 2:** Risk factors for first episode of venous thromboembolism

*Genetic Risk Factors*
Antithrombin deficiency
Protein C deficiency
Protein S deficiency
Factor V Leiden
Prothrombin gene mutation
Non-O ABO blood group
Dysfibrinogenemia
Elevated Factor VIII
Elevated Factor IX
Elevated Factor XI
Hyperhomocysteinemia (including homocystinuria)
*Acquired Risk Factors*
Increasing age
Cancer
Antiphospholipid syndrome
Infections (HIV, Sepsis, etc.)
Inflammatory disorders (e.g. SLE, IBD, vasculitis, etc.)
Nephrotic syndrome
Obesity
Smoking
*Environmental*
Surgery (major inpatient, ambulatory)
Trauma
Immobilization
Central venous catheter
Pregnancy/post-partum
Hormonal therapy (e.g. oral, transcutaneous, vaginal ring contraceptive, Depot progestin injections, hormone replacement, etc.)
Chemotherapy
Travel

**Fig. 2:**
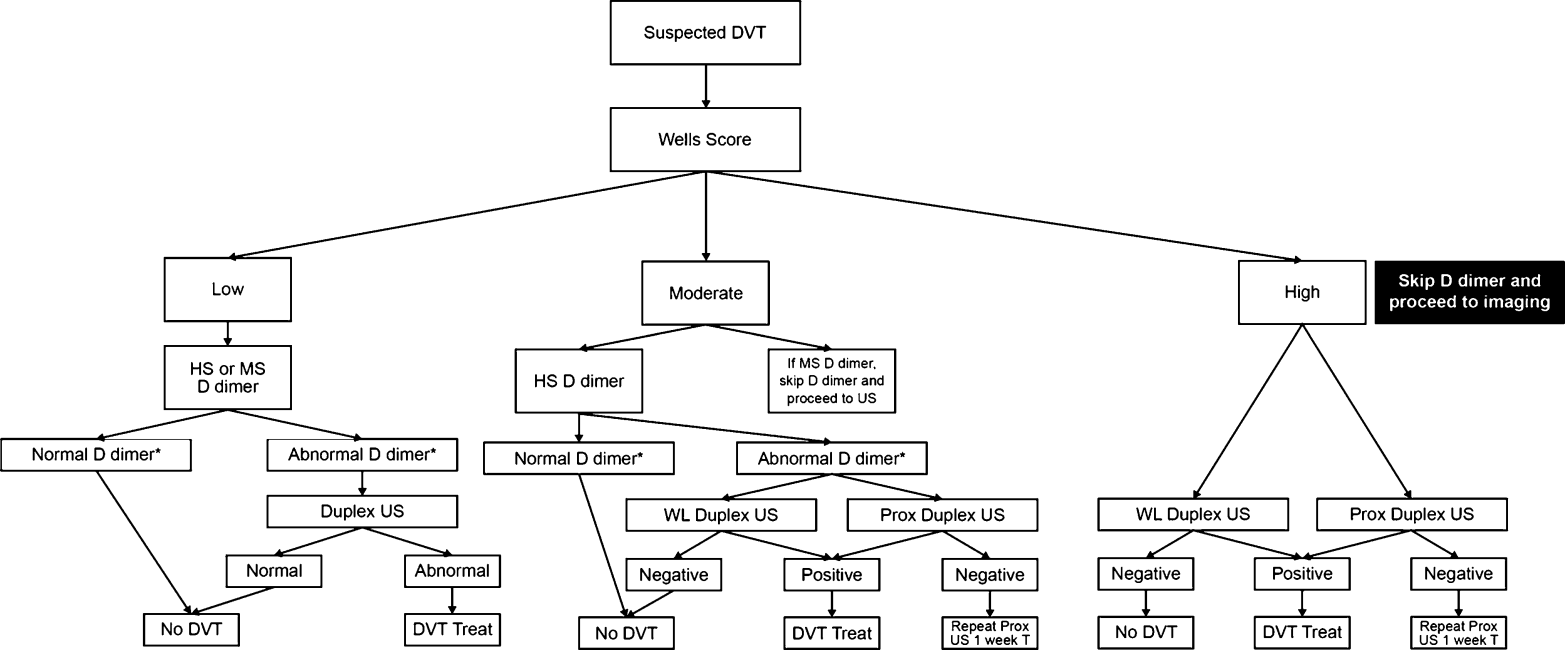
A diagnostic approach to DVT. *HS* High sensitivity, *MS* moderate sensitivity, *US* Ultrasound, *WL* whole leg. High sensitivity D dimer assays include enzyme-linked immunofluorescence assays, microplate enzyme-linked immunosorbent assays (ELISAs) and quantitative latex or immunoturbidimetric assays. Moderate sensitivity assays include whole blood red cell agglutination assays and semiquantitative latex bead agglutination assays. * Using the lab designated threshold for DVT/PE diagnosis NOT the lab normal range for the D dimer assay. If the threshold for DVT/PE diagnosis is not reported by the lab, contact the lab for more information

**Fig. 3:**
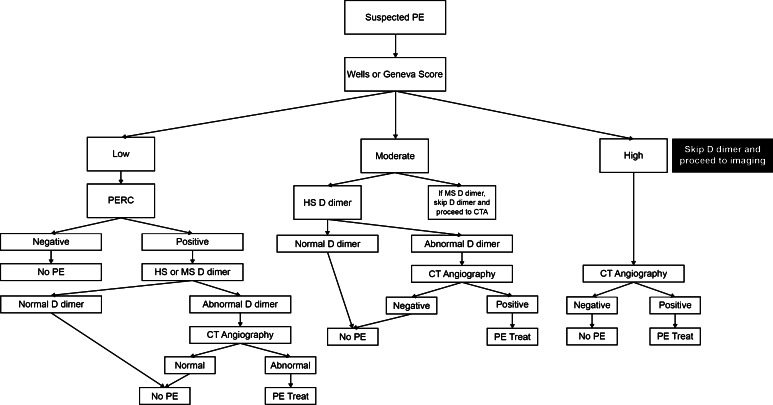
Diagnostic approach to PE. *PERC* Pulmonary Embolism Rule-out Criteria, *HS* High sensitivity, *MS* Moderate sensitivity, *CTA* CT Angiography. High sensitivity D dimer assays include enzyme-linked immunofluorescence assays, microplate enzyme-linked immunosorbent assays (ELISAs) and quantitative latex or immunoturbidimetric assays. Moderate sensitivity assays include whole blood red cell agglutination assays and semiquantitative latex bead agglutination assays. * Using the lab designated threshold for DVT/PE diagnosis NOT the lab normal range for the D dimer assay. If the threshold for DVT/PE diagnosis is not reported by the lab, contact the lab for more information

Pre-test probability models have been developed to facilitate a consistent and structured approach to the diagnosis of VTE. The best studied and validated models are the Wells’ criteria for DVT and PE diagnosis and the Geneva Score for PE diagnosis (Tables 3, 4, 5) [3–5]. In conjunction with D dimer testing, these models have been demonstrated to safely exclude a DVT or PE without use of objective diagnostic imaging in outpatients presenting with suspected VTE. A wide variety of D dimer assays are available on the market for use in VTE diagnosis. Highly sensitive assays include enzyme-linked immunofluorescence assays (sensitivity 96 %; 95 % CI 89–98 %), microplate enzyme-linked immunosorbent assays (ELISAs) (sensitivity 94 %; 95 % CI 86–97 %), and quantitative latex or immunoturbidimetric assays (sensitivity 93 %; 95 % CI 89–95 %). Whole blood red cell agglutination assays (sensitivity 83 %; 95 % CI 67–93 %) and semiquantitative latex bead agglutination assays (sensitivity 85 %; 95 % CI 68–93 %) are considered moderately sensitive D dimer assays. Since the sensitivity of D dimer assays varies considerably, it is important to follow manufacturer recommendations closely when using D dimer assays in the diagnosis of VTE, [6]. The Pulmonary Embolism Rule-out Criteria (PERC) is a clinical decision support tool developed by Kline and coworkers to identify outpatients presenting with chest pain who are thought to be at low risk for PE in whom further diagnostic testing can be avoided (Table 6) [7, 8]. A recent metaanalysis of 12 studies encompassing over 14,000 patients confirmed the accuracy of the PERC [9]. Consequently, the PERC was included in the American College of Physician’s Practice Guideline on the diagnosis of pulmonary embolism [10]. A schematic depiction of the use of the Wells criteria and the Geneva Score in conjunction with the PERC and D dimer testing in the diagnosis of DVT and PE is displayed in Figs. 2 and 3 [11]. A recent patient level meta-analysis of studies using the Wells rule in exclusion of DVT found that in conjunction with a negative D dimer test, the Wells Score was safe and efficient in men and women, both inpatients and outpatients. A notable exception was patients with cancer [12]. Age-adjusted D dimer thresholds have been prospectively demonstrated to increase the efficiency of exclusion of PE without increasing the rate of missed diagnoses. If the diagnosis of DVT or PE is confirmed, treatment is initiated as outlined below [13]. In patients at moderate or high pre-test probability of DVT or PE, if diagnostic testing must be delayed, some experts have recommended that therapy should be initiated until the diagnosis can be confirmed [14].

**Table 3:** Wells clinical DVT model

Clinical characteristic	Score
Active cancer (patient receiving treatment for cancer within 6 months or currently receiving palliative treatment)	1
Paralysis, paresis, or recent plaster cast immobilization of the lower extremities	1
Recently bedridden for 3 days or more, or major surgery within the previous 12 weeks requiring general or regional anesthesia	1
Localized tenderness along the distribution of the deep venous system	1
Entire leg swollen	1
Calf swelling at least 3 cm larger than the asymptomatic side (measured 10 cm below the tibial tuberosity	1
Pitting edema confined to the symptomatic leg	1
Collateral superficial veins (non-varicose)	1
Previously documented deep vein thrombosis	1
Alternative diagnosis at least as likely as deep vein thrombosis	−2

A score of ≤0 indicates that a low pretest probability of deep vein thrombosis. A score of 1 or 2 points indicates a moderate risk of DVT and a score of 3 or higher indicates a high risk of deep vein thrombosis [152]

**Table 4:** Wells clinical pulmonary embolism model

Clinical characteristic	Score
Active cancer (patient receiving treatment for cancer within 6 months or currently receiving palliative treatment)	1
Surgery or bedridden for 3 days or more during the past 4 weeks	1.5
History of deep venous thrombosis or pulmonary embolism	1.5
Hemoptysis	1
Heart rate > 100 beats/min	1.5
Pulmonary embolism judged to be the most likely diagnosis	3
Clinical signs and symptoms compatible with deep venous thrombosis	3

A score of <2 indicates a low probability of pulmonary embolism. A score of 2–6 indicates an intermediate probability of PE. A score of more than 6 indicates a high probability of pulmonary embolism. Kearon C, Ginsberg JS, Douketis J, et al. (2006) An evaluation of D-dimer in the diagnosis of pulmonary embolism: a randomized trial. Ann Intern Med Jun 6; 144(11):812-21

**Table 5:** Revised Geneva Score Pulmonary Embolism Model (Simplified version)

Clinical characteristic	Score
Previous PE or DVT	1
Heart rate	
75-94 beats/min	1
≥ 95 beats/min	2
Surgery or fracture within last month	1
Hemoptysis	1
Active cancer	1
Unilateral lower limb pain	1
Pain on lower limb deep venous palpation and unilateral edema	1
Age > 65 years	1

A score of <2 indicates a low probability of pulmonary embolism. A score of 2–4 indicates an intermediate probability of PE. A score of 5 or more indicates a high probability of pulmonary embolism. Klok FA, Mos IC, Nijkeuter M, et al. (2008) Simplification of the revised Geneva score for assessing clinical probability of pulmonary embolism. Arch Intern Med 168(19):2131–2136

**Table 6:** Pulmonary embolism rule-out criteria

Clinical Characteristic	Meets criterion	Does not meet criterion
Age < 50 years	0	1
Initial heart rate < 100 beats/min	0	1
Initial oxygen saturation >94 % on room air	0	1
No unilateral leg swelling	0	1
No hemoptysis	0	1
No surgery or trauma within 4 weeks	0	1
No history of venous thromboembolism	0	1
No estrogen use	0	1

Pretest probability with a score of 0 is less than 1 %. Derived from Kline JA, Courtney DM, Kabrhel C, et al. (2008) Prospective multicenter evaluation of the pulmonary embolism rule-out criteria. J Thromb Haemost 6(5):772–780

In patients with renal insufficiency in whom intravenous contrast is contraindicated, PE should be evaluated with ventilation perfusion imaging. If non-diagnostic, a negative proximal leg duplex study rules out the diagnosis of PE in patients with a low pre-test probability. In patients at moderate or high pretest probability, additional imaging should be considered to confirm the diagnosis (e.g. whole leg duplex or echocardiography) [14]. In the meantime, treatment should continue until the diagnosis is excluded.

For the diagnostic approach to cancer-associated VTE and pregnant patients with suspected VTE see the papers by Khorana et al. and Bates et al., respectively, in this issue.

### **Guidance Statement**

*We suggest the use of validated pre-test probability models in conjunction with D dimer testing and selective use of objective diagnostic imaging to increase the cost-efficiency and accuracy of VTE diagnosis.*

(2) Which patients require hospitalization versus initial outpatient therapy for the management of VTE?

The availability of LMWH, fondaparinux and direct oral anticoagulants has increased the options for acute outpatient treatment of DVT and PE. Contraindications to outpatient management of DVT and PE are listed in Table 7. Outpatient management of DVT has been compared to inpatient management in six randomized controlled trials that included 1708 participants. These studies found that patients treated at home with LMWH were less likely to suffer recurrent VTE (fixed effect relative risk (RR) 0.61; 95 % confidence interval (CI) 0.42–0.90) and major bleeding (RR 0.67; 95 % CI 0.33–1.36) and had lower mortality (RR 0.72; 95 % CI 0.45–1.15). However, it is important to note that these studies had high exclusion rates and many patients who received outpatient treatment were initially managed as inpatients [15].

**Table 7:** Contraindications to outpatient treatment of venous thromboembolism

Active or high risk of bleeding
Recent surgery (within 7 days)
Cardiopulmonary instability
Severe symptomatic venous obstruction
High risk pulmonary embolism*
Thrombocytopenia (platelets < 50,000/µL)
Other medical or surgical condition requiring inpatient management
Medical non-compliance
Geographical or telephone inaccessibility
Poor hepatic function (International Normalized Ratio (INR) ≥ 1.5)
Unstable renal function (e.g. rising serum creatinine)
Poor home health care support environment

* High risk PE is characterized by systolic blood pressure <90 mmHg or a systolic blood pressure drop of ≥40 mmHg for >15 min not due to an arrhythmia, hypovolemia or sepsis

A number of different approaches have been taken to identify PE patients at low risk for adverse outcomes who might be safely managed as outpatients including use of clinical risk assessment models (PESI, Hestia, Geneva), laboratory biomarkers of right ventricular strain (e.g. troponin, NT pro-BNP) and imaging studies (CT or echocardiogram assessment of right ventricular overload) [16]. The four chamber cardiac view on chest CT can be used to identify right ventricular pressure overload. In a retrospective study of 431 patients with PE, RV enlargement on CT was an independent predictor of 30 day mortality (hazard ratio: 5.17;95 % CI 1.63–16.35) [17]. However, a meta-analysis of 10 studies of normotensive PE patients determined that although CT RVD was associated with an overall increased risk of death (OR 1.8 95 % CI 1.3–2.6), with death resulting from PE(OR 7.4; 95 % CI 1.4–39.5), and with PE-related complications (OR 2.4; 95 % CI 1.2–4.7), CT only demonstrated modest utility in assessing risk for adverse outcomes and thus should not be used in isolation for determining management [18].

Echocardiographic evidence of RV dysfunction has been identified as an independent predictor of adverse outcomes. However, a meta-analysis noted that echocardiography had an unsatisfactory negative likelihood ratio for early all-cause mortality (0.62; 95 % CI 0.41–0.92) and PE-related mortality (0.36; 95 % CI 0.20–0.80). This result may be due to the lack of standardized echocardiographic criteria for RV dysfunction and the difficulty inherent in attempting to differentiate between acute and chronic RV overload [19]. Therefore, it is currently premature to rely upon echocardiography to identify low risk patients with PE.

Several clinical prediction models have been developed to determine the outcome of patients with acute PE including the Pulmonary Embolism Severity Index (PESI) score, the Geneva score and the Hestia criteria (Tables 8, 9, 10). Of these, the PESI score and a simplified version, sPESI, have been the most extensively validated. In a multicenter prospective open randomized clinical trial of inpatient versus outpatient management of low risk PE patients as determined by the PESI score, Aujesky et al. found that there was no difference between outpatients and inpatients in recurrent VTE (1 of 171, 0.6 % vs. 0 of 168; 95 % upper CI limit 2.7 %), major bleeding (3 of 171, 1.8 % vs. 0 of 168, 0 %, 95 % upper CI limit 4.5 %) and 90 day mortality (1 of 171, 0.6 % vs. 1 of 168, 0.6 %; 95 % upper CI limit 2.1 %). These data indicate that outpatient management of low risk PE patients (as identified by the PESI score) is feasible and associated with excellent outcomes [20]. The HESTIA criteria have also been demonstrated to be useful in identifying patients for outpatient management [21].

**Table 8:** Pulmonary embolism severity index (PESI) score

Predictors	Points assigned
Age, years	Age, in years
Altered mental status*	+60
Systolic blood pressure <100 mmHg	+30
History of cancer	+30
Arterial oxygen saturation <90 %^‡^	+20
Temp < 36 °C	+20
Respiratory rate ≥ 30/min	+20
Pulse ≥ 110/min	+20
Male sex	+10
History of heart failure	+10
History of chronic lung disease^†^	+10

A total point score for a given patient is obtained by summing the patient’s age in years and the points for each applicable predictor. Points assignments correspond with the following risk classes: Class 1 (very low risk): ≤65; Class II (low risk): 65–85; Class III (intermediate risk): 86–105; Class IV (high risk): 106–125; Class V (very high risk): >125

^†^ Chronic obstructive pulmonary disease

^‡^ With and without supplemental oxygen administration

* Altered mental status was defined as confusion, disorientation, somnolence, lethargy, stupor, or coma

**Table 9:** Simplified PESI score

Predictors	Points assigned
Age > 80 years	1
History of cancer	1
History of heart failure	1
Pulse > 110 beats/min	1
Systolic blood pressure < 100 mmHg	1
Arterial oxygen saturation < 90 %	1

Low risk = total point score 0

**Table 10:** Hestia criteria

Criteria
Hemodynamically instable (e.g. HR > 100 beats/min, systolic BP < 100 mmHg, needs ICU admission)
Thrombolysis or embolectomy necessary
High risk of bleeding (e.g. GI bleed within 14 days, recent stroke (within 4 weeks), recent surgery (within 2 weeks), platelets < 75,000/µL, uncontrolled HTN (systolic BP > 180 mmHg, diastolic BP > 110 mmHg)
Supplemental O_2_ needed to keep O_2_ saturation > 90 %for > 24 h
Pulmonary embolism during anticoagulation treatment
Intravenous pain medication > 24 h
Medical or social reason for in-hospital treatment > 24 h
Creatinine clearance < 30 mL/min
Severe liver impairment
Pregnant
Documented history of HIT

The presence of any criterion precludes outpatient treatment

Cardiac biomarkers that are released from myocytes during right ventricular strain have also proven useful for identification of PE at risk for adverse outcomes. In a multicenter prospective study of cardiac biomarkers for risk stratification of PE, Vuilleumier and colleagues found that a NT-pro-BNP level < 300 pg/mL had a negative predictive value of 100 % (95 % CI 91–100) for adverse outcomes at 3 months. Troponins have also been identified as useful biomarkers for risk stratification in PE [22]. High sensitivity assays for troponin I and T have also been useful in identification of low risk patients with PE. In a prospective validation study of 526 normotensive patients with PE, Lankeit et al. noted that only 4 of 214 (1.9 %) patients with a high sensitive troponin T < 14 pg/mL had adverse outcomes at 30 days. When combined with a simplified Pulmonary Embolism Severity Index (sPESI) score of zero, none of 127 patients with this combination had adverse outcomes [23]. A combination of clinical and laboratory biomarkers may represent the ideal strategy for identification of normotensive patients at low risk for adverse outcomes. Jimenez et al. conducted a multicenter cohort study of normotensive PE patients to identify a multi-marker prognostic score for risk stratification. The combination of a sPESI and a BNP level <100 pg/mL was associated with a negative predictive value of 99 and 100 % in the derivation and validation cohorts [24].

A recent systematic review of outpatient treatment of PE including 11 studies and 1258 patients noted that the rates of recurrent VTE (1.47 %; 95 % CI 0.47–3.0 %), fatal PE (0.47 %; 95 % CI 0.16–1.0 %), major bleeding (0.81 %; 95 % CI 0.37–1.42 %) and mortality (1.58 %; 95 % CI 0.71–2.80 %) were low, similar to the rates identified in inpatient treatment studies. Furthermore, the authors found that both “clinical gestalt” and standardized risk assessment models appeared to be equally useful in identifying low risk patients appropriate for outpatient management. However, they recommended that future studies comparing formal risk stratification models and “clinical gestalt” should be conducted since there was more heterogeneity in the studies on clinical gestalt [25].

Management of patients with PE should be guided by an assessment of their risk for adverse outcomes (Table 11). Normotensive patients in PESI Class I or II or simplified PESI Class 0 do not need further risk stratification with imaging (e.g. echocardiography) and can be considered for outpatient management. Normotensive patients in PESI Class ≥ II or simplified PESI ≥ 1 should undergo additional imaging and laboratory risk assessment and warrant initial inpatient management until the results of these studies are complete. Patients in this group who have no sign of right ventricular dysfunction on echocardiography or abnormal cardiac biomarkers are considered at low intermediate risk for adverse outcomes. This group of patients can be considered for early discharge from the hospital. Patients with abnormal echocardiography or cardiac biomarkers are consider intermediate-low risk patients and are often managed in the hospital. Patients with abnormal echocardiography and cardiac biomarkers are considered at intermediate high risk of adverse outcomes and are generally managed as inpatients. Intermediate high risk PE patients are considered for thrombolytic therapy on a case-by-case basis. PE patients with hypotension are at high risk for adverse outcomes. They routinely undergo echocardiography and are strongly considered for thrombolytic therapy [14]. Further discussion of PE management can be found in the accompanying paper by Vedantham et al.

**Table 11:** Mortality risk categories for patients with acute pulmonary embolism

30 day mortality risk	Risk factors			
	Hypotension	PESI Class III through V	RV dysfunction	Abnormal cardiac biomarkers
High	Present	Optional assessment	Present	Optional test
Intermediate-high	Absent	Present	Present	Present
Intermediate-low	Absent	Present	Either one or neither present	
Low	Absent	Absent	Absent but test not necessary	Absent but test not necessary

Adapted from: [14]

### **Guidance Statement**

*We suggest that most patients with DVT and many patients with PE can be managed as outpatients. PE patients should be risk stratified to determine appropriate management. A variety of laboratory tests and imaging modalities as well as clinical risk prediction models are available to identify PE patients who are suitable for outpatient management. Further research is needed to identify the optimal approach to risk stratification of PE patients.*

(3) What are the therapeutic options for the acute treatment of venous thromboembolism?

Anticoagulation (AC) is the primary approach to therapy during all three phases of VTE treatment (acute, short term and long term). For those with life- or limb-threatening thrombosis or in patients with significant thrombus burden, systemic (for PE) or catheter-directed thrombolysis in conjunction with mechanical thrombectomy can be considered in the acute phase of treatment. Application of these therapies in the short term treatment phase of therapy is associated with a less favorable benefit:risk ratio as the thrombus becomes better organized and correspondingly less amenable to lysis/fragmentation.

In patients with contraindications to anticoagulation, placement of a vena cava filter can be considered in patients at risk for PE. In patients with distal “calf” DVT, serial duplex studies can be considered to determine if clot extension occurs that would place the patient at risk for PE warranting filter placement if AC is still contraindicated.

The acute treatment phase corresponds to the initial 5–10 days of therapy when parenteral therapy is traditionally used during the transition to vitamin K antagonists which were the primary therapy during the short term and long term phases of therapy for VTE (Fig. 1). The goals during the acute phase are to rapidly extinguish thrombin and fibrin clot generation. Achieving this goal reduces the symptoms associated with acute VTE and prevents thrombus extension and embolization. Prevention of further thrombus formation also allows the body’s fibrinolytic system to begin the process of thrombus dissolution.

**Fig. 4:**
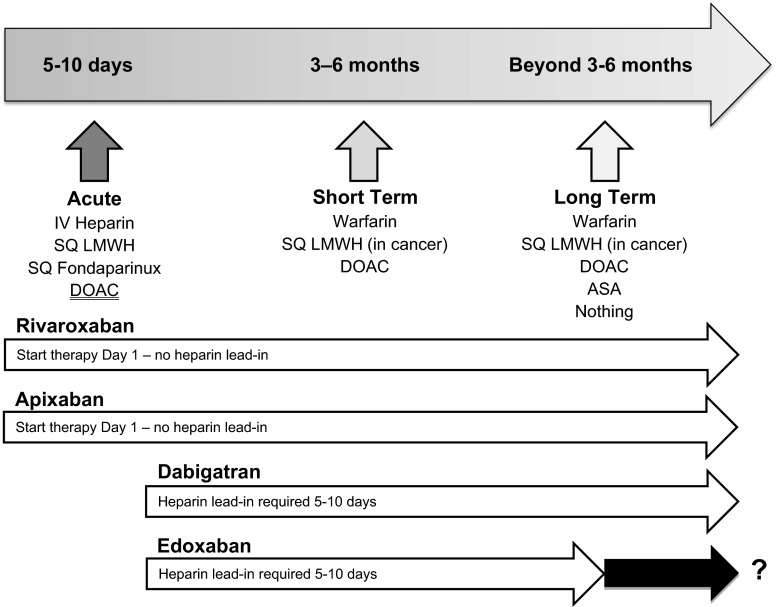
Therapeutic options for anticoagulant treatment of VTE?

For patients with acute VTE who are candidates for anticoagulation, multiple therapeutic options are now available to the clinician (Fig. 4). If the patient is hospitalized, unfractionated heparin (UFH) or low molecular weight heparin (LMWH) are generally utilized given their shorter elimination half-lives that facilitate peri-procedural management (Table 12). In patients felt to be at high bleeding risk, unfractionated heparin may be preferable due to its shorter half-life and complete reversibility. UFH may also be preferable in special patient populations such as morbidly obese (BMI ≥ 40 kg/M^2^) and underweight patients (weight < 50 kg) as well as patients with severe renal impairment or unstable renal function (creatinine clearance <30 mL/min). The disadvantages of intravenous unfractionated heparin are significant inter-individual dose requirements that make close laboratory therapeutic monitoring a necessity. Since the sensitivity of different aPTT reagents to UFH varies substantially, it is important for each laboratory to establish its own therapeutic range based upon UFH levels as measured by protamine titration or chromogenic anti-Xa levels [26]. Observational studies have demonstrated that optimal management of UFH is difficult to achieve in routine clinical practice [27]. In addition, UFH poses an 8-10-fold higher risk for heparin-induced thrombocytopenia (HIT) than LMWH [28, 29].

**Table 12:** Treatment options for VTE

Acute VTE treatment options	Elimination half-life
Unfractionated heparin: 80 U/kg intravenous bolus followed by 18 U/km/h infusion adjusted to activated partial thromboplastin time (aPTT) ratio	1 h
Low molecular weight heparin	
Dalteparin 100 U/kg subcutaneously every 12 h or 200 U/kg subcutaneously every 24 h Renal dosing: no official recommendation-use with caution, consider LMWH anti-Xa levels monitoring and dose adjustment	3–5 h (Half-life 5.7 h after IV administration of 5000 units in hemodialysis patients compared with 2.1–2.3 h in normal renal function)
Enoxaparin 1 mg/kilogram subcutaneously every 12 h or 1.5 mg/kilogram subcutaneously every 24 h FDA approved renal dosing-1 mg/kg sc q24hours (CrCl < 30 mL/min)	4.5–7 h (17 % lower clearance with mild renal impairment-CrCl 50–80 mL/min; 31 % lower clearance with moderate renal impairment-CrCl 30–50 mL/min 44 % lower with severe renal impairment-CrCl < 30 mL/min)
Tinzaparin 175 U/kg subcutaneously every 24 h Renal dose: same (no evidence of bioaccumulation in the IRIS study)	3-4 h (24 % reduced clearance in severe renal impairment-CrCl < 30 mL/min)
Pentasaccharide	
Fondaparinux 5–10 mg subcutaneously every 24 h (5 mg for weight <50 kg, 7.5 mg for weight 50–100 kg and 10 mg for weight > 100 kg) Renal dosing: Avoid in patients with CrCl < 30 mL/min; caution in patients with CrCl 30–50 mL/min	17–21 h (25 % lower clearance with mild renal insufficiency-CrCl 50–80 mL/min; 40 % lower with moderate renal impairment-CrCl 30–50 mL/min; 55 % lower with severe renal impairment-CrCl < 30 mL/min)
Direct oral anticoagulants	
Apixaban (oral direct factor Xa inhibitor) 10 mg orally BID X 7 days then 5 mg po BID In patients with at least 2 of the following characteristics: age ≥ 80 years, body weight ≤60 kg, or serum creatinine ≥1.5 mg/dL, the recommended dose is 2.5 mg orally BID. Would avoid in patients with CrCl < 25 mL/min or sCr > 2.5 mg/dL or hepatic dysfunction (AST/ALT > 2 × ULN or bilirubin > 1.5X ULN)	12 h
Dabigatran (oral direct thrombin inhibitor) 150 mg orally BID after 5–10 days of initial parenteral anticoagulation (Avoid in patients with CrCl < 30 mL/min and liver impairment with transaminase > 2x ULN))	13 h (CrCl ≥ 80 mL/min) 15 h (CrCl 50–79 mL/min) 18 h (CrCl 30–49 mL/min) 27 h (CrCl 15–29 mL/min)
Edoxaban (oral direct factor Xa inhibitor) 60 mg orally once daily 30 mg once daily if CrCl 15–50 mL/min or body weight ≤60 kg or Avoid in patients with CrCl < 15 mL/min or Child-Pugh class B/C hepatic impairment	10–14 h (Total systemic exposure increased by 32 % (CrCl 50–79 mL/min), 74 % (30–49 mL/min), 72 % (CrCl < 30 mL/min), and 93 % (peritoneal dialysis), respectively)
Rivaroxaban (oral direct factor Xa inhibitor) 15 mg orally BID X 3 weeks followed by 20 mg once daily Avoid in patients with CrCl < 30 mL/min and Child-Pugh class B/C	5–9 h (age 20–45 years) 11–13 h (age ≥ 65 years)
Vena cava filter	

Given the disadvantages associated with UFH, LMWH is often preferred outside of special hospitalized patient populations. Fondaparinux can also be employed as a parenteral agent for hospitalized patients in whom transition to a vitamin K antagonist (VKA) is anticipated. A distinct advantage for fondaparinux is an extremely low incidence of HIT. However, fondaparinux has several limitations as an anticoagulant for inpatients including its long half-life (17–21 h with normal renal function) and lack of an antidote [26]. Detailed information about the pharmacology and clinical use of UFH, LMWH and fondaparinux can be found in the accompanying papers by Nutescu et al. and Smythe et al.

If a VKA is anticipated to be the agent for the short term phase of treatment, initiation of VKA therapy should be delayed until all planned invasive procedures are completed and the patient has resumed regular oral intake. If these conditions are satisfied, VKA therapy can begin as soon as therapeutic levels of UFH/LMWH are achieved. Parenteral therapy with UFH or LMWH should continue for at least 5 days of overlap and until an INR of 2 or more is achieved for 24 h. Both these goals should be achieved before discontinuation of parenteral therapy [30]. Detailed information about warfarin dosing and its management can be found in the accompanying paper by Witt et al.

Direct oral anticoagulants (DOACs) are also an option for the treatment of VTE in hospitalized patients. While DOACs are advantageous because they do not require monitoring, they are not easily reversible, have longer elimination half-lives (7–15 h) than UFH or LMWH and could accumulate in patients with suboptimal renal (estimated creatinine clearance <30 mL/min) or hepatic function (Child-Pugh class B or C). In addition, experience with perioperative management is limited. Therefore, DOACs are optimized for outpatient rather than inpatient use [31]. If either dabigatran or edoxaban are chosen, therapy must include 5 days of parenteral anticoagulation prior to beginning these agents. In contrast, rivaroxaban and apixaban can both be used for acute treatment of VTE without initial parenteral therapy.

Thrombolytic therapy is an important management option in patients with acute extensive proximal lower extremity DVT or patients with proximal DVT that fails to respond to initial anticoagulation. Catheter-directed pharmacomechanical thrombolysis/thrombectomy is typically employed in patients with acute (within 2 weeks) proximal (ilio-femoral) deep vein thrombosis at significant risk for long term post-thrombotic complications or poor outcomes with conventional anticoagulation who are at low risk for bleeding complications. May-Thurner syndrome (MTS) (iliac vein compression syndrome) is a congenital anatomic alteration in which the left iliac vein is compressed between the right iliac artery and the lumbosacral spine. Compression results in intravascular strictures that slow venous flow which may precipitate thrombus formation [32]. Consequently, catheter-directed pharmacomechanical thrombolysis and thrombectomy in conjunction with angioplasty and venous stenting has been advocated to reduce the risk of recurrent thrombosis although well designed studies supporting this contention are lacking [33]. Further investigation in this area is warranted. Until these data are available, patients with May-Thurner syndrome associated iliac vein deep venous thrombosis should be managed on a case-by-case basis. Irrespective of interventional management, therapeutic anticoagulation is required.

In patients with PE, systemic thrombolytic therapy is generally reserved for patients with massive pulmonary embolism (i.e. high risk pulmonary embolism with systemic hypotension and right ventricular dysfunction). Thrombolytic therapy is applied in a case-by-case basis in patients with sub-massive PE (i.e. intermediate risk pulmonary embolism in normotensive patients with right ventricular dysfunction) who are at low risk for bleeding complications. Catheter-based and surgical thromboembolectomy are other options available to providers for patients with hemodynamically significant PE [14]. A complete discussion of thrombolytic therapy for PE and DVT can be found in the accompanying paper by Vedantham et al.

### **Guidance Statement**

*With the variety of treatment options available, we recommend that the acute therapy of VTE should be customized to suit the unique clinical circumstances of the individual patient. We suggest that unfractionated heparin may be preferable for inpatients with planned invasive procedures, recent major bleeding episodes or severely impaired renal function as well as underweight and morbidly obese patients although several members of panel felt there were insufficient data to support this suggestion. LMWHs are convenient options for inpatient and outpatient therapy. DOACs are optimized for outpatient therapy of VTE.*

We suggest that systemic and catheter-directed pharmacomechanical thrombolytic therapy are effective options for treatment of massive PE and acute extensive proximal DVT that can rapidly reduce thrombus burden. Given the greater risks of bleeding associated with these approaches, we recommend that a careful assessment of the risks and benefits of therapy should be performed in each patient prior to the initiation of thrombolytic therapy.

(4) Which patients are candidates for a DOAC?

Direct oral anticoagulants offer a convenient and attractive approach to the treatment of VTE since they are oral, do not require routine laboratory monitoring and have fewer drug–drug interactions than oral VKA. DOACs have been demonstrated to be at least as effective as conventional treatment for VTE. However, patients with poor renal and/or hepatic function, pregnancy/breast feeding, thrombocytopenia, high bleeding risk and potent drug–drug interactions were excluded from participation in the phase 3 VTE studies. In addition, certain patient populations were not well represented in these studies such as patients with active cancer. Therefore, it is important to consider the inclusion and exclusion criteria and the enrolled populations in the published studies when considering a DOAC for treatment of VTE. In addition, 2 of the DOACs (dabigatran and edoxaban) were studied using acute treatment with a parenteral agent (dabigatran median duration 9 days; edoxaban median duration 7 days). Therefore, these agents should be used only after an initial period of parenteral therapy for acute VTE (Fig. 4).

*Dabigatran* is an oral direct thrombin inhibitor that has been compared to warfarin in the short term treatment and warfarin and placebo in long term treatment of VTE in 3 double blind randomized controlled trials, the RECOVER, REMEDY and RESONATE studies. In the RE-COVER study, 2564 patients with acute symptomatic objectively documented proximal lower extremity DVT or PE were randomized to either dabigatran 150 mg twice daily or adjusted-dose warfarin (INR range 2–3) after acute treatment with unfractionated or low molecular weight heparin (median parenteral treatment duration = 9 days). Seven patients in the dabigatran group and 18 in the warfarin group did not receive study medication leaving a total of 1274 dabigatran patients and 1265 warfarin patients in the population for efficacy analysis. In the warfarin group, the time in therapeutic range over the duration of the study was 60 % (53 % month 1, 66 % in the last month). Thirty of 1274 patients on dabigatran (2.4 %) and 27 of 1265 warfarin recipients (2.1 %) suffered recurrent VTE (0.4 % absolute risk difference; 95 % CI for non-inferiority −0.8 to 1.5). The hazard ratio (HR) with dabigatran was 1.10 (95 % CI 0.65–1.84). Major bleeding occurred in 20 patients assigned to dabigatran (1.6 %) and in 24 patients taking warfarin (1.9 %) for a hazard ratio with dabigatran of 0.82 (95 % CI 0.42–1.48) (Table 13). There was no difference in mortality, acute coronary events or abnormal liver function tests [34].

**Table 13:** Results of randomized controlled trials of DOACs versus conventional therapy for VTE

Study	Treatment	Patients	Recurrent VTE^1^	Major bleeding
RE-COVER, 2009	Dabigatran 150 mg BID vs. VKA	1273/1266	30 (2.4 %) vs. 27 (2.1 %) (HR 1.10; 95 % CI −0.8 to 1.5)	20 (1.6 %) vs. 24 (1.9 %) (HR 0.82; 95 % CI 0.45–1.48)
RE-COVER II, 2014	Dabigatran 150 mg BID vs. VKA	1279/1289	30 (2.3 %) vs. 28 (2.2 %) (HR 1.08; 95 % CI 0.64–1.80)	15 (1.2 %) vs. 22 (1.7 %) (HR 0.69; 95 % CI 0.36–1.32)
EINSTEIN DVT, 2010	Rivaroxaban 15 mg BID X 3 weeks, then 20 mg daily vs. Enoxaparin/VKA	1731/1718	36 (2.1 %) vs. 51 (3.0 %) (HR 0.68; 95 % CI 0.44–1.04)	14 (0.8 %) vs. 20 (1.2 %) (HR 0.65; 95 % CI 0.33–1.30)
EINSTEIN PE, 2012	Rivaroxaban 15 mg BID X 3 weeks, then 20 mg daily vs. Enoxaparin/VKA	2419/2413	50 (2.1 %) vs. 44 (1.8 %) (HR 1.12; 95 % CI 0.75–1.68)	26 (1.1 %) vs. 52 (2.2 %) (HR 0.49; 95 % CI 0.31–0.79)
AMPLIFY, 2013	Apixaban 10 mg BID X 7 days then 5 mg BID vs. Enoxaparin/Warfarin	2609/2635	59 (2.3 % vs. 71 (2.7 %) (RR 0.84; 95 % CI 0.60–1.18)	15 (0.6 %) vs. 49 (1.8 %) (RR 0.31; 95 % CI 0.17–0.55)
HOKUSAI-VTE, 2013	Edoxaban 60 mg daily (or 30 mg daily) vs. warfarin	4118/4122	130 (3.2 %) vs. 146 (3.5 %) (HR 0.89; 95 % CI 0.70–1.13)	56 (1.4 %) vs. 66 (1.6 %) (HR 0.84; 0.59–1.21)

^1^Recurrent VTE primary endpoint was symptomatic VTE and VTE-related death in the RE-COVER and RE-COVER II studies, the AMPLIFY study and the HOKUSAI-VTE study. In the EINSTEIN studies it was recurrent symptomatic VTE

These results were confirmed in RECOVER II, a randomized double-blind double dummy study that compared dabigatran 150 mg twice daily with warfarin (INR 2–3) after median of 9 days of parenteral therapy. Recurrent symptomatic objectively confirmed VTE occurred in 30 of 1279 dabigatran patients (2.3 %) and 28 of 1289 warfarin patients (2.2 %) (HR 1.08; 95 % CI 0.64–1.80). Major bleeding occurred in 15 dabigatran patients (1.2 %) and 22 warfarin patients (1.7 %) (HR 0.69; 95 % CI 0.36–1.32) (Table 13). Pooled analysis with the RECOVER and RECOVER II studies produced a hazard ratio for recurrent VTE of 1.09 (95 % CI 0.76–1.57), major bleeding of 0.73 (95 % CI 0.48–1.11) and for any bleeding of 0.70 (95 % CI 0.61–0.79) [35]. These studies demonstrate that dabigatran is at least as effective as warfarin for short term treatment of VTE. Compared with warfarin, dabigatran was associated with an increased risk of myocardial infarction or acute coronary syndrome in a meta-analysis of the randomized clinical trials (RCT) leading to its approval (dabigatran, 237 of 20,000 [1.19 %] vs. control, 83 of 10,514 [0.79 %]; OR 1.33; 95 % CI 1.03–1.71; P = 0.03) [36]. However, no difference was seen in a recent large new user cohort of 134,414 propensity-matched elderly US Medicare patients (Hazard ratio 0.92 (95 % CI 0.78–1.08) perhaps due to clinical differences in the two study populations [37]. Until this issue is further clarified, prescribers should use caution when prescribing dabigatran in elderly patients at risk for acute coronary syndrome.

GI bleeding also appears to be more common in higher risk patients treated with dabigatran compared with warfarin. The risk of GI bleeding with dabigatran was significantly higher than warfarin in the RE-LY RCT in atrial fibrillation (RR 1.30; 95 % CI 1.07–1.56) but similar in VTE (RECOVER RR1.79; 95 % CI 0.60–5.32; RECOVER II 0.60; 95 % CI 0.22–1.66; REMEDY RR 0.62; 95 % CI 0.22–1.90) [38]. This difference likely reflects differences in study populations as the AF patients tended to be older and/or on concomitant antiplatelet agents more commonly than VTE patients. This interpretation is borne out by a new-user Medicare cohort of AF patients in which dabigatran was associated with an increase in major GI bleeding (RR 1.28 (95 % CI 1.14–1.44) [37]. It is important to note that there is a dose-related difference in the risk of GI bleeding between dabigatran 150 mg twice daily (Relative risk 1.50; 95 % CI 1.19–1.89) and 110 mg twice daily (RR 1.10; 95 % CI 0.86–1.41) [39]. Only the 150 mg dose is available in the United States. Given the GI bleed data from the RE-LY study, it may be worthwhile considering another DOAC than dabigatran for older patients with VTE.

### **Guidance Statement**

*When used after a 5–10 day initial course of parenteral anticoagulation, dabigatran is as effective as warfarin in the acute and short term treatment of VTE. We suggest dabigatran as an alternative to vitamin K antagonists for the short term therapy of VTE. In some studies, dabigatran has been associated with an increased risk of acute coronary syndrome and gastrointestinal bleeding compared with vitamin K antagonists.*

*Rivaroxaban*, an oral direct factor Xa inhibitor, has been compared with conventional therapy for acute, short term and long term treatment of VTE in the EINSTEIN DVT and PE trials as well as the EINSTEIN Extension trial [40, 41]. In contrast to the dabigatran VTE studies, the EINSTEIN DVT and PE trials were open-label event driven randomized controlled trials. Patients were randomized within 48 h of diagnosis to either conventional therapy (enoxaparin transitioned to adjusted dose warfarin or acenocoumarol INR 2–3) or rivaroxaban 15 mg twice daily for 3 weeks followed by 20 mg once daily. The median duration of enoxaparin in the EINSTEIN DVT study was 8 days and 80.8 % of patients had an INR of 2 or more at the end of treatment. The overall time in therapeutic range was 57.7 % (54.1 % in month 1 and 66.4 % in month 10). Recurrent VTE occurred in 36 rivaroxaban patients (2.1 %) and 51 enoxaparin/VKA patients (3.0 %) (HR 0.68; 95 % CI 0.44–1.04). Major bleeding occurred in 14 rivaroxaban patients (0.8 %) and 20 enoxaparin/VKA patients (1.2 %) (HR 0.65; 95 % CI 0.33–1.30). The principal safety outcome (major or clinically relevant non-major bleeding) was also similar between groups (rivaroxaban, 139 [8.1 %] vs. enoxaparin/VKA 138 [8.1 %]; HR 0.97 [95 % CI 0.76–1.22]) [40]. The EINSTEIN PE trial had a similar design to the EINSTEIN DVT trial. The median duration of enoxaparin therapy in the enoxaparin/VKA arm was 8 days and 83 % of patients achieved an INR of 2.0 or more by the end of enoxaparin treatment. The time in therapeutic range for VKA patients over the course of the study was 62.7 % (57.8 % during the first month and 72.7 % during month 11). Symptomatic recurrent VTE occurred in 50 patients taking rivaroxaban (2.1 %) and 44 patients who received enoxaparin/VKA (1.8 %) (HR 1.12; 95 % CI 0.72–1.68). Major or clinically relevant non-major bleeding occurred in 249 rivaroxaban patients (10.3 %) and 274 (11.4 %) enoxaparin/VKA patients (HR 0.90; 95 % CI 0.76–1.07). Major bleeding occurred in 26 rivaroxaban patients (1.1 %) and 52 enoxaparin/VKA patients (2.2 %) (HR 0.49; 95 % CI 0.31–0.79) (Table 13). These studies demonstrate that rivaroxaban is a safe and effective alternative for acute and short term therapy of VTE. Major bleeding was similar or lower with rivaroxaban compared with conventional therapy. No increase in gastrointestinal bleeding or acute coronary events was seen. However, patients age 75 and older appear to be at increased risk of GI bleeding with rivaroxaban compared with warfarin, therefore caution is warranted in these patients [38, 42, 43].

### **Guidance Statement**

*Rivaroxaban is as effective as LMWH/VKA in the treatment of DVT and PE. We suggest rivaroxaban as an alternative to LMWH/VKA for the acute and short term treatment of VTE in appropriate patients. No increase in acute coronary syndrome has been seen with rivaroxaban, however GI bleeding may be more common in patients age 75 and older.*

*Apixaban*, an oral direct factor Xa inhibitor, was compared to conventional therapy (enoxaparin followed by warfarin) for treatment of VTE in the AMPLIFY study and to placebo in the AMPLIFY EXT trial [44, 45]. Similar to the EINSTEIN studies, patients in the AMPLIFY study were enrolled within 36 h of diagnosis and apixaban was started immediately without initial parenteral therapy. In contrast to the EINSTEIN studies, the AMPLIFY study had randomized double-blind double-dummy study design. Apixaban was administered in an initial dose of 10 mg twice daily for 7 days followed by 5 mg twice daily for 6 months. The duration of enoxaparin therapy in the conventional treatment arm was 6.5 days. For warfarin patients, the INR was in the therapeutic range 61 % of the time during the study. Recurrent VTE occurred in 59 of 2609 apixaban patients (2.3 %) and 71 of 2635 conventional therapy patients (2.7 %) (Relative Risk (RR) 0.84; 95 % CI 0.60–1.18). Major bleeding occurred in 15 of 2676 apixaban patients (0.6 %) and 49 of 2689 conventional therapy patients (1.8 %) (RR 0.31; 95 % CI 0.17–0.55). Major or clinically relevant non-major bleeding was also lower in apixaban-treated patients (4.3 %) than conventional therapy patients (9.7 %) (RR 0.44; 95 % CI 0.36–0.55). All-cause mortality was similar between groups (1.5 % vs. 1.9 %; RR 0.79; 95 % CI 0.53–1.19) [44] (Table 13). These results indicate that apixaban, like rivaroxaban is an attractive one drug treatment for acute and short term therapy of VTE compared to conventional therapy. No increase in acute coronary events was seen compared to warfarin [42].

### **Guidance Statement**

*Apixaban is as effective as LMWH/VKA in the treatment of DVT and PE and associated with less major bleeding and major or clinically relevant non-major bleeding. We suggest apixaban as an alternative to LMWH/VKA in the acute and short term treatment of VTE in appropriately selected patients. No increase in acute coronary syndrome or gastrointestinal bleeding has been seen with apixaban.*

*Edoxaban* is a direct oral inhibitor of factor Xa that is capable of inhibiting free and bound factor Xa. Edoxaban was compared with warfarin in the treatment of VTE in the HOKUSAI-VTE study, a large randomized double-blind non-inferiority study conducted in 8292 patients enrolled in 439 centers in 37 countries [46]. After a median of 7 days of parenteral therapy (unfractionated or low molecular weight heparin) following enrollment, patients were randomized to edoxaban 60 mg once daily (30 mg once daily if creatinine clearance 30–50 mL/min, body weight of 60 kg or less or concomitant therapy with a potent P-glycoprotein inhibitor) or placebo and warfarin or matching placebo. A total of 4921 patients had a DVT and 3319 had PE. Extensive thrombus burden (common femoral vein or iliac vein DVT or PE with involvement of multiple lobes with 25 % or more of the entire pulmonary vasculature) was present in 743 (45 %) edoxaban patients and 778 (46.6 %) warfarin patients. Right ventricular dysfunction was noted in 172 edoxaban PE patients (34.5 %) and 179 warfarin PE patients (35.5 %). Recurrent symptomatic VTE occurred in 130 (3.2 %) edoxaban patients and 146 (3.5 %) warfarin patients (HR 0.89; 95 % CI 0.70–1.13). Among patients who qualified for the edoxaban 30 mg daily dose, recurrent VTE occurred in 22 of 733 (3.0 %) edoxaban patients and 30 of 719 (4.2 %) warfarin patients (HR 0.73; 95 % CI 0.42–1.26). The rate of recurrent symptomatic VTE in patients with PE and right ventricular strain was 3.3 % in edoxaban patients and 6.2 % in warfarin patients (HR 0.52; 95 % CI 0.28–0.98). The primary safety outcome (major or clinically-relevant non-major bleeding) occurred in 349 (8.5 %) edoxaban patients and 423 (10.3 %) warfarin patients (HR 0.81; 95 % CI 0.71–0.94). Major bleeding occurred in 56 (1.4 %) edoxaban patients and 66 (1.6 %) warfarin patients (HR 0.84; 95 % CI 0.59–1.21). Among patients who fulfilled criteria for the 30 mg edoxaban dose, 58 of 733 (7.9 %) edoxaban patients and 92 of 719 (12.8 %) warfarin patients (HR 0.62; 95 % CI 0.44–0.86) developed clinically relevant non-major bleeding [46] (Table 13). The Hokusai VTE study confirms that once daily edoxaban is as effective as warfarin in the prevention of recurrent VTE and caused significantly less bleeding following an initial course of parenteral therapy.

### **Guidance Statement**

*After an initial 5–10 days of LMWH or UFH, edoxaban is as effective as LMWH/VKA in the treatment of acute DVT and PE but associated with less major or clinically relevant non-major bleeding. We suggest edoxaban as an alternative to VKA for the short term treatment of VTE in appropriately selected candidates.*

(5) What is the role of vena cava filters if the patient is not a candidate for anticoagulation?

The only reason to consider placement of an inferior vena cava filter is acute VTE (within 4 weeks) in the presence of a contraindication to anticoagulation (i.e. the presence of active bleeding or the presence of risk factors for major bleeding (e.g. recent major bleeding event, major surgery or major trauma, etc.) [47]. Other indications are controversial and of unproven clinical benefit or are frankly harmful. As with any procedure it is important to assess whether the risks of a vena cava filter are warranted by its benefits on a case-by-case basis. Potential complications of a vena cava filter are indicated in Table 14. In general we do not suggest vena cava filters for distal lower extremity DVT, superficial venous thrombophlebitis, VTE older than 1 month, or upper extremity DVT. In the case of upper extremity DVT, the risks of symptomatic and fatal PE are low and the severity of potential complications of filter thrombosis in the superior vena cava or penetration of thoracic vascular structures by filter struts or during the insertion procedure exceed the benefits [48].

**Table 14:** Complications of inferior vena cava filters

Access site thrombosis
Deep venous thrombosis
Filter migration/embolization
Filter misplacement (outside target zone)
Filter strut fracture
Guidewire entrapment
IVC thrombosis
IVC penetration
Pulmonary embolism
Inability to remove retrievable filter

In the event of a recent surgical procedure, the timing of initiation of anticoagulation varies according to the bleeding risks posed by the surgical procedure (Table 15). The timing of anticoagulation outlined in the table should not be considered proscriptive; rather it should be considered a rough guide for practice. It is better to err on the side of caution and wait a few extra days to initiate anticoagulation even if it means placing a retrievable vena cava filter as post-operative bleeding can result in significant complications and further delays in treatment. It is recommended that active filter follow up programs be instituted so patients do not get lost to follow up. These programs have a high rate of success with filter retrieval (>95 %) [49]. These decisions should be based upon local expertise and experience.

**Table 15:** Risk stratification of bleeding risk with anticoagulation following surgery

Bleeding risk category	Type of surgery or procedure	Anticoagulation recommendation
Very high	Neurosurgical procedure (intracranial or spinal) Prostatectomy or partial nephrectomy, bladder surgery Heart valve replacement Coronary artery bypass grafting	Can initiate prophylactic dose anticoagulation at 24 h Consider therapeutic dose anticoagulation no sooner than 72 h
High	Pacemaker or AICD placement Major cancer surgery Major vascular surgery (AAA repair, peripheral artery bypass) Reconstructive plastic surgery Renal or hepatic biopsy Bowel polypectomy (assume this will be part of a colonoscopy) Major orthopedic surgery	Can initiate prophylactic dose anticoagulation within 12–24 h Consider therapeutic dose anticoagulation no sooner than 48–72 h
Moderate	Major intra-abdominal surgery Major intra-thoracic surgery	Can initiate prophylactic dose anticoagulation within 12–24 h Consider therapeutic dose anticoagulation no sooner than 24–48 h
Low	Laparoscopic cholecystectomy or hernia repair Coronary angiography Arthroscopy Biopsy (prostate, bladder, thyroid, lymph node) Bronchoscopy ± biopsy Central venous catheter removal Multiple dental extraction or gum surgery	Can initiate prophylactic dose anticoagulation within 12 h Consider therapeutic dose anticoagulation 24–48 h
Very low	Minor dental procedures (single tooth extractions or root canals) (See Table 6) Minor dermatologic procedures (excisions of basal and squamous cell carcinomas, actinic keratoses, and malignant or premalignant nevi) Cataract removal Electroconvulsive therapy (ECT) Arthrocentesis Joint or soft tissue injections GI endoscopy without biopsy	Interruption of anticoagulation typically not necessary

Decisions on initiation of anticoagulation should always include a discussion with all members of the care team including the operating surgeon. In high risk bleeding situations, we suggest use of unfractionated heparin initially and starting the infusion without a bolus. Once patients are therapeutic for at least 24 h without evidence of bleeding, they can be transitioned to a more convenient agent on a case by case basis depending upon the preferences of the care team. If the severity of the thrombotic event dictates use of a bolus, the risks of bleeding that might be associated with its administration must be balanced with the risks associated with a vena cava filter.

In the event of a gastrointestinal bleed, we suggest waiting at least 7 days without evidence of active bleeding and after endoscopic treatment of the bleeding lesion before reinitiating therapeutic anticoagulation [50]. In the event of intracranial hemorrhage (ICH), it is essential to review the indications for anticoagulation and the patient’s risk of recurrent VTE as recurrent ICH is common (2.56 per 100 patient years) and potentially deadly (25 % case fatality rate) [51]. In general, only patients with recent VTE (within 3 months), idiopathic VTE or VTE with ongoing potent risk factors (active cancer, lupus anticoagulant positive APS, etc.) or recurrent unprovoked VTE warrant consideration of resumption of anticoagulation. In addition, one must factor in the risk of rebleeding. Lobar ICH is associated with a higher risk of recurrence than deep hemispheric bleeds [52]. Underlying diseases or lesions associated with the initial hemorrhage should be treated prior to resumption of anticoagulation. The optimal time to resume anticoagulation remains uncertain but a recent large retrospective cohort study of warfarin-associated ICH suggested that resumption of warfarin between 10 and 30 weeks was associated with the lowest risk of recurrent ICH and thromboembolism. While only 30 of the 177 patients who survived the first week had VTE as an indication for anticoagulation, only 4 of these patients (13 %) suffered recurrent VTE and none were fatal. In contrast, 18 patients suffered recurrent ICH (10 %) of which 4 were fatal (22 %). Although these data are imperfect with respect to management of patients with VTE, they indicate that only the highest risk VTE patients should consider resumption of anticoagulation after a spontaneous ICH [53].

Once a patient has successfully resumed therapeutic anticoagulation without recurrent bleeding complications, we refer them back to the interventional radiologist who placed their vena cava filter. The latest generation of retrievable vena cava filters can be retrieved with a high degree of success six or more months after placement. Therefore, it may be preferable to wait several months before retrieving the filter in order to make sure the patient will tolerate anticoagulation. In patients with filters that cannot be retrieved, the impact of this on anticoagulation duration needs to be considered. In patients with transient indications for anticoagulation, the risks of thrombosis associated with a vena cava filter need to be balanced against the risks of bleeding associated with anticoagulation. In the PREPIC study, 36.4 % suffered a DVT and 14 % suffered IVC thrombosis after 8 years of follow up [54].

### **Guidance Statement**

*We suggest that vena cava filters should be considered in any patient with acute VTE (within 4 weeks) who cannot be treated with anticoagulation. We suggest that retrievable filters are strongly preferred as most patients have temporary contraindications to anticoagulation. Filters should be retrieved once anticoagulation can be reinitiated preferably within 6 months of placement. Patients with filters should be closely monitored in a structured program to facilitate retrieval and minimize the number of patients lost to follow up.*

Following anticoagulation-associated gastrointestinal bleeding, we suggest that anticoagulation can be re-initiated as early as 7 days after cessation of bleeding and treatment of causal lesions. Following anticoagulation associated ICH, we suggest resumption of anticoagulation no sooner than 10 weeks post-bleed. Further investigation of this topic is warranted.

(6) How is upper extremity VTE treated?

Upper extremity DVT is often associated with an intrinsic or extrinsic precipitant. The most common extrinsic precipitant is the presence of a central venous catheter (CVC), pacemaker/implanted cardiac defibrillator or venous intervention. In these cases, the DVT originates at the location of the device/intervention. If the DVT is anatomically distant from the catheter or pacemaker then other reasons should be sought [55]. In patients with a CVC-associated DVT, anticoagulation alone without CVC removal is successful in many patients and allows preservation of the CVC for continued use in the event that an indication for central venous access remains avoiding the morbidity associated with the insertion of a new CVC [56]. If symptoms fail to improve after initial anticoagulation, then the CVC can be removed [55]. Although there are no data as to when the risk of PE with CVC removal in patients with CVC-associated DVT declines, a meta-analysis of recurrent VTE in randomized controlled treatment trials of VTE suggests that delaying removal for at least 1 week will greatly reduce the risk of PE associated with CVC removal [57]. If the patient is not a candidate for anticoagulation, CVC removal rather than placement of a superior vena cava filter is recommended given the hazards associated with filter thrombosis or strut penetration in this location and the lower risk of PE associated with upper extremity DVT [58]. The duration of AC therapy for CVC-associated DVT/PE should be at least 3 months or as long as the CVC remains in place. A similar approach to duration of therapy can be taken in cancer patients with CVC associated VTE [15, 59].

In patients with an upper extremity DVT associated with pacemakers or implanted defibrillators, anticoagulation without device removal is the primary approach to management [60]. In a prospective study, risk factors for thrombosis included hormonal therapy, a history of VTE and an absence of anticoagulant treatment. Of these, only hormonal therapy and an absence of anticoagulation remained significant in multivariate analysis [61]. No treatment studies have been performed in this patient population but the authors of this guidance document suggest at least 3 months of anticoagulation is appropriate.

In patients with an upper extremity DVT in the absence of a CVC, an anatomic trigger should be considered. In younger patients with upper extremity DVT, the presence of thoracic outlet syndrome (TOS) or effort induced thrombosis (Paget-von Schroetter) syndrome (PSS) should be investigated. Thoracic outlet syndrome occurs when the nerve, artery and/or vein traversing the thoracic outlet are compressed by the surrounding anatomic structures. This compression can cause venous, arterial or neurologic compromise. In the venous form of this syndrome, the compression causes endothelial damage and stasis leading to local anatomic clot formation. In PSS repetitive upper extremity exercise usually in the context of a tight thoracic outlet can lead to vascular damage, stasis and subsequent thrombus formation. A history of upper extremity thrombosis in the absence of CVC or upper extremity or thoracic or neck vein intravenous access procedures should prompt consideration of TOS. A recent history of upper extremity exertion or exercise should also raise suspicions of TOS/PSS. Patients often complain of aching and swelling in the upper extremity and demonstrate venous distention and bluish discoloration in the affected arm. Physical exam findings that suggest the presence of TOS include Adson’s test (ipsilateral rotation and extension of the neck during deep inspiration result in a diminution of the radial pulse) and Wright’s test (hyperextension of the arm diminishes the radial pulse). Nevertheless, imaging studies are essential to demonstrate TOS. Venous and arterial duplex ultrasound with the patient’s arm in stress positions are the most sensitive study for assessing the presence of TOS [15, 59].

For TOS/PSS, thrombolytic therapy followed by surgical repair (thoracic rib resection and/or scalenectomy) has been advocated as an important component of successful therapy in addition to anticoagulation. Thrombolysis followed by endovascular stenting does not appear to be as beneficial as surgery [62]. However, the benefits of surgical therapy remain to be demonstrated in a rigorous fashion [63]. Consequently, the writing committee of this guidance document was divided as to the value of surgical repair of thoracic outlet syndrome. Until well-designed studies are conducted examining the risks and benefits of surgical therapy, we suggest providers consider surgical repair in addition to thrombolysis and anticoagulation versus thrombolysis/anticoagulation alone on a case-by-case basis. We suggest that patients with upper extremity deep vein thrombosis receive at least 3 months of anticoagulation with or without surgical therapy.

Other important causes of upper extremity DVT include intra-thoracic or cervical tumors or nodal masses or infections that can result in vascular wall inflammation and compression. Diagnosis can generally be established with duplex ultrasound or contrast CT venographic imaging. Identification and treatment of the underlying disease process (cancer, infection, etc.) and anticoagulation are both likely to be important factors in successful treatment. We suggest that anticoagulation should be continued for at least 3 months or until precipitating factors have been eliminated (vascular compression by tumor), whichever is longer [64].

### **Guidance Statement**

*Identification and elimination of trigger factors when feasible is important to reduce the incidence of recurrent upper extremity DVT. For CVC-associated DVT, we suggest that anticoagulation without CVC removal is the treatment of choice. If symptoms fail to resolve, CVC removal can be considered. We suggest that anticoagulation should be continued for at least 3 months or the duration of the CVC whichever is longer. At least 3 months of anticoagulation is appropriate for pacemaker wire-associated VTE.*

The committee was divided as to the optimal approach to treatment of TOS/PSS-associated upper extremity DVT. The benefits of rib resection/scalenectomy following thrombolysis and anticoagulation remain to be rigorously demonstrated. Therefore, providers should consider therapy for TOS/PSS on a case-by-case basis until higher quality data are available. We suggest that TOS/PSS-associated upper extremity DVT warrants anticoagulation for at least 3 months. Treatment of upper extremity DVT associated with extrinsic compression due to cancer or infection should include treatment of the underlying disease in addition to anticoagulation.

(7) When is ambulation/exercise safe after DVT/PE?

Four randomized controlled trials have examined the question of whether early ambulation with or without early compression therapy is associated with an increased risk of pulmonary embolism. Patients began to walk on the day of diagnosis (3 studies) or after 2 days of leg elevation. No difference on symptomatic pulmonary embolism was noted (risk ratio 1.16 [95 % CI 0.66–2.05]). Early ambulation was associated with a reduction in acute limb pain and an improvement in quality of life due to DVT in one study (p < 0.01 and p < 0.05) whereas it had no effect in two other studies. Early ambulation did not increase the risk of thrombus progression (RR 0.38; 95 % CI 0.13–1.15) [65]. Although the number of patients is small, the existing literature suggest that ambulation in patients with acute DVT/PE is safe as soon as therapeutic anticoagulation is achieved.

### **Guidance Statement**

*We suggest that ambulation is safe in patients with acute DVT ± PE after achievement of therapeutic anticoagulation.*

(8) Is the use of graduated compression stockings safe after acute DVT/PE?

In the 4 ambulation studies mentioned above, 2 studies prescribed compression therapy to both groups and 2 studies applied compression therapy only in the ambulation arm so we cannot use these studies to determine the safety of GCS during the acute treatment of VTE. Brandjes et al. randomized 194 patients to knee high GCS or no stockings for prevention of post-thrombotic syndrome within 2–3 weeks of a first episode of DVT. No difference in the rate of recurrent VTE was noted between the groups (14 of 96 patients [14·6 %] in the GCS group vs. 13 of 98 patients in no GCS group [13·3 %) [66]. Similarly, Prandoni et al. found no difference in recurrent VTE in their open randomized study of GCS for prevention of PTS (GCS group 12/90 [13.3 %] vs. control group 13/90 [14.4 %]). The average time of enrollment was 7 days (range 5–10 days) after diagnosis [67]. Finally the SOX trial, a double blind placebo controlled randomized controlled trial of GCS in the prevention of PTS also noted no difference in recurrent VTE between active GCS patients (33 patients [8·1 %]; 45 events [36 DVT, 9 pulmonary embolism]) and placebo stocking patients (38 patients [9·6 %]; 44 events [32 DVT, 12 pulmonary embolism]. The median time to enrollment was less than 5 days [68]. This study also showed that GCS did not significantly reduce leg pain associated with DVT [69]. These studies indicate that application of GCS during the acute treatment of VTE is not associated with an increased risk of recurrent VTE but does not appear to reduce pain associated with acute DVT. For further discussion of graduated compression stockings, see the accompanying paper by Kahn et al.

### **Guidance Statement**

*We suggest that GCS do not increase the risk of recurrent thromboembolism in patients with acute VTE. We suggest that GCS do not have any beneficial effect on leg discomfort associated with acute DVT*.

(9) What is the recommended duration of therapy for VTE?

Long term therapy corresponds to anticoagulation beyond 3–6 months when the primary goals of therapy are to continue to suppress thrombin generation in order to prevent recurrent VTE. The primary long term treatment of VTE is anticoagulation. In the past, oral VKA were the mainstay of long term therapy except for in cancer patients in whom LMWH has been preferred. DOACs also represent an attractive option for long term therapy of VTE in appropriate candidates. In patients with contraindications to initial anticoagulation, vena cava filters are employed. In these patients, it is important to routinely reassess patients for the continued presence of contraindications to anticoagulation on an ongoing basis as VCF are associated with an increased risk of recurrent DVT and IVC thrombosis. Since the majority of patients have temporary contraindications to anticoagulation, retrievable filters with a broad window of retrievability should be used. In patients on long term anticoagulation it is important to reassess the risks and benefits of continued anticoagulation on a routine basis given the changing medical circumstances of patients over time.

- What is the recommended duration of therapy for a patient with distal (calf vein) DVT?

Distal (calf vein) DVT represents venous thrombosis that occurs in the distal deep veins of the lower extremity which include the paired anterior and posterior tibial veins and the peroneal veins as well as the muscular veins of the calf (gastrocnemius and soleus). The consequences of distal DVT are less than those of proximal DVT and PE so different management options exist. The risks of distal DVT include extension to the proximal deep veins, PE and post-thrombotic syndrome. Early studies of distal DVT noted that 20 % of calf vein DVT extend into the proximal deep vein system, primarily within 1 week of diagnosis [70, 71]. However, more recent studies have found lower rates of extension. MacDonald et al. found that only 3 % of patients with distal DVT experience proximal extension of thrombosis [72]. PE also appears to occur less frequently with calf vein DVT. In the CALTHRO study, only one of 64 patients with calf DVT suffered PE in 3 months of follow up [73]. In contrast, in the population-based observational Worchester VTE study, the rate of recurrent VTE (30 days: 7.6 vs. 4.1 %; 6 months: 11.0 vs. 8.7 %; 1 year: 11.0 vs. 11.5 %; P = 0.47) and PE (30 days: 1.9 vs. 1.0 %, 6 months: 2.6 vs. 1.8 %;1 year 3.3 vs. 2.4 %; P = 0.72) was similar among patients with distal and proximal DVT although use of anticoagulation or vena cava filters was less common (No anticoagulation or IVC filter; 15.9 vs. 7.7 %; p = 0.01) [74]. In the RIETE multicenter prospective registry, recurrent DVT (24/1921, 1.3 % vs. 135/9165, 1.5 %, Odds ratio (OR) 0.85 [0.55–1.31], p 0.45) and PE (14/1921, 0.7 % vs. 114/9165, 1.2 %; OR 0.58 [0.33–1.02], p = 0.06) were similar between distal and proximal DVT, although recurrent PE approached significance. Use of anticoagulation for 10 or more days was similar (96.8 vs. 97.3 %, p = 0.24) between distal and proximal DVT although patients with distal DVT were less likely to be treated for 3 months (89.1 vs. 91.8 %, p < 0.001) or receive an IVC filter (0.7 vs. 1.8 %, p < 0.001) [75]. In the OPTIMEV study cohort, patients with distal and proximal DVT suffered recurrent VTE (17 of 787, 2.2 % vs. 15 of 598, 2.5 %) at similar rates (OR 0.8 [0.4–1.8]) [76]. In a prospective open randomized clinical trial, Lagerstedt et al. found that patients with symptomatic distal DVT that received 5 days of heparin only had a much higher rate of progressive/recurrent thrombosis than patients who received heparin followed by 3 months of warfarin (8/28 vs. 0/23, p < 0.01). Seven of the 8 patients had symptomatic recurrence. This study has been criticized for its small size and open label design and the use of an antiquated surveillance strategy, ^99m^technetium labeled plasmin scanning [77]. The open label randomized pilot study, the Anticoagulation of Calf Thrombosis (ACT), also found suggestive evidence of a difference in outcomes with anticoagulation versus symptomatic treatment with non-steroidal anti-inflammatory medications and acetaminophen. No patient in the anticoagulation arm (N = 35) suffered progressive thrombosis compared with 4 of 35 patients (11.4 %, 95 % CI −1.5 to 26.7 %, p = 0.11) in the no anticoagulation arm [78].

Although clearly more data are needed, we favor anticoagulation for the acute treatment of symptomatic isolated calf vein DVT. In patients deemed to be at high risk for bleeding with anticoagulation, we favor repeat duplex studies in 1 week for evidence of proximal extension over placement of a vena cava filter. Risk factors for thrombus extension include an elevated D dimer, extensive thrombosis (e.g. length > 5 cm, maximal diameter >7 mm, multiple veins involved) or thrombus closed to the proximal deep veins, unprovoked thrombosis, active cancer, a history of VTE and inpatient status. Conversely, thrombosis involving only the calf muscles (e.g. soleus, gastrocnemius) appear to be at lower risk of progression [72].

There is limited information on the duration of therapy for patients with isolated distal DVT. Pinede et al. conducted an open randomized trial of 6 versus 12 weeks of anticoagulation. The risk of recurrent VTE (2 of 105, 2 % vs. 3 of 92, 3.4 %; Relative risk (RR) 0.58 [0.10–3.36]) and major bleeding (1 of 105, 1 % vs. 3 of 92, 3.4 %; RR 0.29 [0.03–2.72]) were similar between the 6 week and 12 week groups [79]. Although this result would favor shorter duration treatment for distal DVT, it should be considered preliminary since this study was terminated early after less than half the target subject population had been recruited due to slow accrual. Therefore, we would suggest 3 months of therapy for patients with distal DVT.

### **Guidance Statement**

*We suggest treatment of distal (calf vein) DVT with anticoagulation versus observation. We suggest a duration of therapy of 3 months. In patients with contraindications to anticoagulation, we favor repeat duplex surveillance in 1 week rather than vena cava filter insertion.*

(b) What is the recommended duration of therapy for a patient with a surgically-provoked VTE?

Major surgery and trauma are major situational triggers for VTE. In the Million Women’s Study, major inpatient surgery was associated with a 70-fold relative risk (95 % CI 63–76) of VTE compared to the general population without surgery during the first 6 post-operative weeks. Ambulatory surgery is associated with a relative risk of VTE of 9.6 (8.0–11.5) in the first 6 weeks post-operation. The relative risks remain substantial for the first 12 weeks and do not return to baseline until 12 months post-operation [80]. Given the potency of these situational thrombotic risk factors, the duration of therapy for anticoagulation is limited to 3 months. In a meta-analysis of randomized controlled trials and prospective observational studies of at least 3 months of anticoagulation for VTE, the risk of recurrence after discontinuation of AC was only 0.7 % per patient-year, which is less than the risk of major bleeding associated with VKA as well as direct oral anticoagulants [81]. Therefore, longer durations of anticoagulation are likely to be associated with net harm. Although previous studies have focused primarily on inpatient surgical procedures, the risk associated with ambulatory surgery is still substantial and transient such that a limited duration therapy is still appropriate. Further studies looking specifically at this population are warranted.

### **Guidance Statement**

*We suggest that 3 months of anticoagulation is adequate for surgical risk factor-associated VTE unless risk factors for recurrence persist.*

(c) What is the appropriate duration of therapy for a pregnancy or estrogen-associated VTE?

Pregnancy is associated with a 4–5 fold increased risk of VTE. The risk of VTE is highest in the early post-partum period but the risk remains elevated up to 6–12 weeks post-partum [82, 83]. In the absence of thromboprophylaxis, women with a previous history of pregnancy-associated VTE have a 2–10 % chance of suffering a recurrent event during pregnancy (OR 24.8; 95 % CI 17.1–36.0) [84–86]. Women with pregnancy-associated VTE have a lower risk of recurrence than women with unprovoked VTE (5.8 vs. 10.4 %, HR 0.6; [95 % CI 0.4–0.9]; p = 0.02). However, women with pregnancy-associated VTE have a higher risk of recurrence during pregnancy than women with a previous unprovoked VTE (4.5 vs. 2.7 %, RR = 1.7, 95 % CI 1.0–2.8) [87]. Therefore, patients with pregnancy-associated VTE should be treated with anticoagulation for at least 3 months and for the duration of the pregnancy and post-partum period (up to 12 weeks post-partum), whichever is longer. During subsequent pregnancies, we recommend that patients should be strongly considered for thromboprophylaxis. The appropriate intensity of thromboprophylaxis remains to be determined, however, recurrent events have occurred in some patients treated with prophylactic doses of LMWH [88].

Hormonal contraceptives and hormone replacement therapy increase the risk of VTE to a varying degree (2–4 fold) depending upon the dose of estrogen, the type of progestin in combined estrogen/progestin tablets and, in the case of hormone replacement therapy, the route of administration [89]. The risk of VTE appears to be higher for oral contraceptive preparations containing gestodene, desogestrel, drospirenone, and cyproterone than those containing levonorgestrel or norgestimate [90]. In the prospective United Kingdom National Health Service population-based Million Women Study, transdermal estrogen only hormone replacement therapy was not associated with an increased risk of VTE (Relative risk 0.82; 95 % C 0.64–1.06) compared to oral estrogen-progestin (RR 2.07; 95 % CI 1.86–2.31] and oral estrogen-only therapy (RR 1.42; 95 % CI 1.21–1.66) [91]. In contrast, in comparison with non-users, users of transdermal combined estrogen-progestin contraceptive patches (RR 7.9; 95 % CI 3.5–17.7) and vaginal rings (RR 6.5; 95 % CI 4.7–8.9) were associated with an increased risk of VTE compared with women who used progestin-only etonogestrel only subcutaneous implants (RR 1.4; 95 % CI 0.6–3.4) and the levonorgestrel intrauterine system (RR 0.6; 95 % CI 0.4–0.8) [92]. In patients with hormone-associated VTE, the risk of recurrence is lower among patients who discontinue hormonal therapy. In the MEGA study the risk of recurrence was 9.7 per 1000 patient-years (95 % CI 4.3–21.5) among patients who completed at least 3 months of anticoagulation and discontinued hormonal therapy. Recurrence rates were higher among patients who continued on hormonal therapy (27.3 per 1000 patient-years [95 % CI 14.7–50.7]), particularly if one focused only on the time period of hormone administration (55.3 per 1000 pt.-years (95 % CI 29.8–102.9). The investigators did not note a difference in recurrence rates between women with or without hormonal therapy exposure prior to their initial VTE (9.7 per 1000 patient-years [4.3–21.5] vs. 16.2 per 1000 patient-years (8.7–30.2)). [93] In the PREVENT study, women with a hormone associated event had a 46 % lower recurrence risk (HR 0.54; 95 % CI 0.19–1.54) [94]. In a prospective cohort study of 660 women with VTE, Eischer et al. noted an adjusted relative risk of 0.4 (95 % CI 0.2–0.8) among estrogen-containing contraceptive users compared with non-users [95]. These findings are corroborated in a patient level meta-analysis by Douketis et al. who found that women with hormone-associated VTE had a 50 % lower risk of recurrence than women without hormone-associated VTE (HR 0.5; 95 % CI 0.3–0.8). When the type of hormonal therapy was specified, women with oral contraceptive-associated VTE had a lower risk of recurrence than non-hormone users (HR 0.39, 95 % CI 0.16–0.91). The risk of recurrence among users of hormone replacement therapy was slightly less although not significant (HR 0.76, 95 % CI 0.39–1.49) [96]. Use of hormones at the time of the index VTE was also associated with a significant reduction in recurrence in the DASH cohort [97]. In patients with a hormone-associated VTE and two negative D dimer results (one on therapy and the second 1 month after discontinuing anticoagulation), the risk of recurrent VTE was very low (0 %, 95 % CI 0.0–3.0 %) [98]. Therefore, if hormonal therapy is medically necessary, anticoagulation should be continued as this strategy has been shown to be effective in preventing recurrent VTE in patients on hormonal therapy [99].

For an in depth discussion of pregnancy-associated VTE see the accompanying paper by Bates et al.

### **Guidance Statement**

*We suggest that patients with pregnancy-associated VTE should be treated for the duration of the pregnancy and the post-partum period (up to 12 weeks post-partum) or as long as dictated by the thrombotic event, whichever is longer. Patients with pregnancy-associated VTE are at high risk for recurrent VTE with subsequent pregnancies, therefore we suggest that thromboprophylaxis for the duration of the pregnancy and post-partum period should be strongly considered.*

Patients with hormone-associated VTE appear to be at lower risk for recurrent VTE particularly if their D dimer is negative at the end of therapy and 1 month after discontinuing anticoagulation. Therefore, we suggest that long term anticoagulation beyond 3–6 months may not be associated with a favorable risk: benefit balance in patients with hormone-associated VTE if hormonal therapy has been discontinued. If hormonal therapy is medically necessary, we suggest that anticoagulation should be continued as these patients are at high risk for recurrent VTE.

(d) What is the recommended duration of therapy for a medical illness-associated VTE?

The presence of an active medical illness at the time of the index VTE is associated with an intermediate risk of recurrent VTE once a course of AC has been completed (4.2 % per patient-year) [81]. Consequently, it is appropriate to consider continuation of anticoagulation for as long as the medical illness is active (i.e. inflammatory bowel disease, nephrotic syndrome, etc.) or at least 3 months, whichever is longer. The identifiable risk factors present at the time of the event should be eliminated prior to discontinuation of anticoagulation to reduce the risk of recurrence. Further research in this area is needed to refine the approach to duration of therapy in this patient population.

### **Guidance Statement**

*We suggest that patients with medical-illness associated VTE should be treated for at least 3 months or as long as the medical risk factors for VTE remain present.*

(e) What is the recommended duration of therapy for a travel-associated VTE?

Travel is a highly publicized risk factor for VTE that is associated with a variable risk of thrombosis. Travel by airplane, car, bus and train all increase the risk of VTE [100]. The duration of travel is the most important risk factor. Regarding air travel, duration of 4–8 h and 8–12 h increase in the incidence of VTE by 2 fold while travel of 12–16 h and >16 h are associated with incidence rate ratios of 5.3 (95 % CI 2.3–12.4) and 5.7 (95 % CI 2.0–16.5), respectively. The risk for VTE associated with travel remains significantly elevated only for 4 weeks post-travel [101]. Therefore, VTE should only be ascribed to air travel if it occurs within this period. Events occurring later are probably better considered as being unprovoked for the purposes of determining the duration of therapy. The risk of air travel-associated VTE is increased by the presence of other concomitant risk factors [102] (see accompanying paper by Heit et al. Therefore, it is important to eliminate any removable risk factors if possible to reduce the chances of recurrent VTE after discontinuation of anticoagulation. Patients with air travel-associated VTE should be treated for at least 3 months. In patients who have suffered air travel-associated VTE, it is reasonable to consider travel prophylaxis. Low molecular weight heparin has been used for this purpose [103]. The most appropriate agent and dose for thromboprophylaxis is unknown. However DOACs are an attractive alternative as they are convenient, easy to administer and effective for prevention of thrombosis in high risk patients and are not associated with the complexities of travelling with syringes that LMWH requires.

### **Guidance Statement**

*Travel (>4 h duration) is a modest and transient risk factor for VTE. Therefore, VTE should only be ascribed to air travel if it presents within 4 weeks of travel and is not associated with other concomitant triggers. In the absence of other precipitants, we suggest that travel-associated VTE should be treated for at least 3 months. We suggest that travel thromboprophylaxis be considered for future travel in these patients.*

(f) What is the recommended duration of therapy for malignancy-associated VTE?

Active cancer is associated with a 4–6 fold increased risk of VTE [104, 105]. This risk is modified by the primary site, type and extent of cancer and its treatment and concomitant pre-existing risk factors for VTE (e.g. thrombophilia). Patients with active cancer are at high risk for recurrent VTE as long as the cancer is present or under active treatment. Therefore, long term anticoagulation is indicated for as long as the cancer is present or under treatment. A risk model for assessing the risk of recurrent VTE in cancer patients has recently been developed [59]. These topics are covered in more detail in the accompanying paper by Khorana et al.

### **Guidance Statement**

*Active cancer is a potent risk factor for VTE that varies with the type and extent of cancer and its treatment. Therefore, we suggest anticoagulation be continued as long as the underlying cancer is active or under treatment.*

(g) What is the recommended duration of therapy for a patient with an unprovoked DVT/PE?

Patients with unprovoked VTE represent the subpopulation of patients at highest risk for recurrent thromboembolism. To qualify for this designation, a patient cannot have another identifiable trigger that contributed to the thrombotic event (e.g. surgery, trauma, medical illness, exogenous hormones, etc.). The presence of unprovoked VTE signifies that this patient is thrombophilic regardless of the identification of a defined thrombophilic state (i.e. factor V Leiden) on laboratory testing. Numerous randomized controlled clinical trials of duration of therapy for VTE have demonstrated that protection against recurrent VTE in patients with unprovoked VTE afforded by anticoagulation only lasts as long as anticoagulation therapy continues. Once anticoagulation is discontinued, the risk of recurrent VTE returns [106–109]. The meta-analysis of Iorio and colleagues estimated that the risk of recurrent VTE among patients with unprovoked VTE is 7.4 % per patient-year, which exceeds the risk of major bleeding posed by anticoagulation in most patients [81]. Therefore, extended anticoagulation is often recommended for patients with unprovoked VTE as intermediate durations of anticoagulation (6 months, 12 months, etc.) have not been associated with lasting reductions in recurrent VTE [15, 110]. Long term therapy trials with DOACs indicate that these medications will be very effective for long term therapy of VTE in patients with unprovoked VTE [40, 45, 111]. In patients with unprovoked VTE who are considering discontinuation of anticoagulation, D dimer testing and multicomponent risk stratification models can be useful to identify patients who are at higher risk for recurrence (see section below).

### **Guidance Statement**

*Patients with unprovoked VTE are at high risk for recurrence so we suggest long term anticoagulation. As there is limited information on the risks and benefits of anticoagulation beyond 2 years, we suggest that providers reassess patients on long term anticoagulation on an annual basis.*

(10) What are the therapeutic options for long term treatment of DVT/PE?

- Vitamin K antagonists

Data from randomized controlled clinical trials of different durations or intensities of anticoagulation support the efficacy and safety of VKA for the long term treatment of VTE. In patients with unprovoked VTE, standard intensity (INR 2–3) anticoagulation is associated with an 88 % relative risk reduction (RR 0.12; 95 % CI 0.05–0.25) of recurrent VTE compared with no treatment. Low intensity VKA therapy (INR 1.5–2) is associated with a 64 % reduction in the risk of recurrent VTE (95 % CI 23–83 %). Anticoagulation with VKA is associated with a 2.6 fold increase in the risk of major bleeding (95 % CI 1.02–6.78) [112] (Table 16). In a double blind randomized controlled trial, standard intensity (INR 2–3) and low intensity (1.5–2) were associated with a similar risk of major bleeding (0.9 per 100 patient-years vs. 1.1 per 100 person-years) [113]. Therefore, it is important to balance the risks and benefits of therapy when considering long term anticoagulation. The case fatality rate for a recurrent episode of VTE has been estimated at 3.6 % while a major bleed is associated with a case fatality rate of 11.3 %. It is important to note that the estimates for the case fatality rate for major bleeding are based upon the initial course of anticoagulation when bleeding tends to be more frequent [114]. These limitations underscore that it is essential to discuss the risk and benefits of therapy with patients to accommodate individual patient preferences.

**Table 16:** Results of randomized controlled trials of long term therapy for VTE

Study	Treatment	Subjects	Recurrent VTE	Major bleeding
LAFIT, 1999	Placebo vs. Warfarin (INR2–3)	83/79 Unprovoked VTE	17 (27 %/pt.-year) vs. 1 (1.3 %/pt.-year.) (HR 0.05; 95 % CI 0.01–0.37)	0 vs. 3 (3.8 %/pt.-year)
PREVENT, 2003	Placebo vs. Warfarin (INR 1.5–2)	253/255 Unprovoked VTE	37 (7.2/100 pt.-year) vs. 14 (2.6/100 pt.-year) (HR 0.36; 95 % CI 0.19–0.67)	2 (0.4/100 pt.-year) vs. 5 (0.9/100 pt.-year) (HR 2.53; 95 % CI 0.49–13.03)
ELATE, 2003	Warfarin (INR 1.5–2) vs. warfarin (INR 2–3)	369/369 Unprovoked VTE	16 (1.9/100 pt.-year) vs. 6 (0.7/100 pt.-year) (HR 2.8; 95 % CI 1.1–7.0)	9 (1.1/100 pt.-year) vs. 8 (0.9/100 pt.year) (HR 1.2;95 % CI 0.4–3.0)
PADIS-PE, 2015	Warfarin (INR2–3) vs. Placebo	184/187 Unprovoked PE	3 (1.7 %) vs. 25 (13.5 %) (HR 0.15; 95 % CI 0.05–0.43)	4 (2.2 %) vs. 1 (0.5 %) (HR 3.96; 95 % CI 0.44–35.89)
RE-MEDY, 2013	Dabigatran 150 mg BID vs. Warfarin (INR2–3)	1430/1426	26 (1.8 %) vs. 18 (1.3 %) (HR 1.44; 95 % CI 0.78–2.64)	13 (0.9 %) vs. 25 (1.8 %) (HR 0.52; 95 % CI 0.27–1.02)
RE-SONATE, 2013	Dabigatran 150 mg BID vs. Placebo	681/662	3 (0.4 %) vs. 37 (5.6 %) (HR 0.08; 95 % CI 0.02–0.25)	2 (0.3 %) vs. 0
EINSTEIN-Extension, 2010	Rivaroxaban 20 mg daily vs. Placebo	602/594	8 (1.3 %) vs. 42 (7.1 %) (HR 0.18; 95 % CI 0.09–0.39)	4 (0.7 %) vs. 0
AMPLIFY-EXT, 2013	Apixaban 5 mg BID or 2.5 mg BID vs. Placebo	840/813/829	Apix 5 mg 14 (1.7 %) vs. Apix 2.5 mg 14 (1.7 %) vs. Placebo 73 (8.8 %) Apix 5 mg vs. Placebo (RR 0.20; 95 % CI 0.11–0.34) or (RR 0.19; 95 % CI 0.11–0.33)	Apix 5 mg 1 (0.1 %) vs. Apix 2.5 mg 2 (0.2 %) vs. Placebo 4 (0.5 %) Apix 5 mg vs. Placebo (RR 0.25; 95 % CI 0.03–2.24) or Apix 2.5 mg vs. Placebo (RR 0.49; 95 % CI 0.09–2.64)
WARFASA, 2012	Aspirin 100 mg daily vs. Placebo	205/197	28 (6.6 % per year) vs. (11.2 % per year) (HR 0.58; 95 % CI 0.36–0.93)	1 vs. 1
ASPIRE, 2012	Aspirin 100 mg daily vs. Placebo	411/411	57 (4.8 % per year) vs. 73 (6.5 % per year) (HR 0.74; 95 % CI 0.52–1.05)	14 vs. 8

### **Guidance Statement**

*Adjusted dose vitamin K antagonists (INR 2–3) reduce the relative risk of recurrent VTE by 88 %, but they are associated with 2.6 fold increase in major bleeding compared with placebo. Consequently, it is important to assess the risks and benefits of long term anticoagulation on a case-by-case basis. Since low intensity (INR 1.5–2) anticoagulation is associated with a similar risk of major bleeding, we prefer standard intensity anticoagulation for long term therapy of VTE.*

(b) LMWH/Fondaparinux

LMWH has been compared with VKA in 15 randomized controlled trials reviewed by Andras et al. in a recent Cochrane review. Recurrent VTE occurred during active therapy in 86 of 1652 patients receiving VKA (5.2 %) and 69 of 1545 patients receiving LMWH (4.5 %) resulting in a non-significant difference between the two treatments (odds ratio (OR) symptomatic recurrent VTE 0.82 [95 % CI 0.59–1.39). There was no evidence of heterogeneity. In pooled analysis the incidence of major bleeding during therapy was 49 of 1652 patients taking VKA (3.0 %) and 24 of 1545 patients taking LMWH (1.6 %) which was associated with a statistically significant difference in favor of LMWH (OR 0.50; 95 % CI 0.30–0.79). No heterogeneity was identified. Mortality was similar between patients taking VKA (59 of 1652, 3.6 %) and LMWH (61 of 1545, 3.9 %) (OR 1.06; 95 % CI 0.74–1.54) [115]. These data demonstrate that LMWH is equivalent to VKA for prevention of recurrent VTE but is associated with fewer major bleeding episodes. No data exist comparing LMWH with placebo for VTE treatment. Although osteoporotic fractures have been seen with UFH therapy in pregnant women [116], no decrease in bone mineral density or osteoporotic fractures have been noted in small studies of pregnant women receiving LMWH [117, 118]. Other disadvantages of LMWH compared to VKA include the necessity for once or twice daily injections, higher costs and a small risk of heparin-induced thrombocytopenia.

Fondaparinux has not been extensively studied for the short term or long term treatment of VTE. Shetty et al. conducted a single arm observational cohort study of fondaparinux in patients with intolerance of VKA therapy. Twenty-six patients completed a 90 day treatment regimen. Sixteen (53 %) had a history of recurrent VTE, eleven (37 %) had idiopathic VTE and 3 (10 %) had cancer. No episodes of recurrent VTE or major bleeding occurred [119]. Pesavento and colleagues reviewed the experience of the RIETE investigators with sub-acute fondaparinux therapy for VTE. Of 47,378 patients in RIETE, 263 were treated for at least 3 months with fondaparinux. Seventy-eight of these patients had cancer. After propensity score matching, there was no difference in recurrent DVT or PE in patients taking fondaparinux and VKA or LMWH. Major bleeding was similar between cancer patients taking fondaparinux (1, 1.2 %) and LMWH (2, 0.65 %), however major bleeding was more common among patients without cancer taking fondaparinux (6, 3.24 %) compared to patients taking VKA (7, 0.95 %) [120]. In conclusion, there are limited data to assess the outcome of patients taking sub-acute or extended fondaparinux for treatment of VTE. Its use in patients without cancer may be associated with a higher risk of bleeding complications. In vitro studies suggest that fondaparinux is likely to be associated with a low risk of osteoporosis [121, 122].

### **Guidance statement**

*Evidence indicates that LMWH is as effective as VKA in the reduction of recurrent VTE but associated with a reduced risk of major bleeding. Limited experience with fondaparinux in the long term treatment of VTE suggests that it is as effective as LMWH in the prevention of recurrent VTE. Fondaparinux may cause more bleeding than VKA in patients without cancer. We suggest that LMWH and fondaparinux are acceptable alternatives to VKA for treatment of VTE.*

(c) Direct Oral Anticoagulants

*Dabigatran* was compared in 2 double-blind randomized clinical trials with warfarin and placebo, respectively for long term treatment of VTE. In the RE-MEDY study, dabigatran 150 mg twice daily was compared with warfarin (INR 2–3) for long term treatment of VTE in patients who had completed at least 3 months of therapy. The median time in therapeutic range for the warfarin group was 65.3 %. Recurrent VTE occurred in 26 of 1830 patients (1.8 %) in the dabigatran group and 18 of 1426 patients (1.3 %) in the warfarin group (Hazard ratio (HR) with dabigatran 1.44; 95 % CI 0.78–2.64). Major bleeding occurred in 13 dabigatran patients (0.9 %) and 25 warfarin patients (1.8 %) (HR 0.52; 95 % CI 0.27–1.02) Acute coronary syndrome occurred in 13 dabigatran patients (0.9 %) and 3 warfarin patients (0.2 %) (p = 0.02). No difference in mortality or liver toxicity was seen. Dyspepsia was noted in 3 % of dabigatran patients. In the placebo-controlled RE-SONATE study, recurrent VTE occurred in 3 of 681 dabigatran patients (0.4 %) compared with 37 of 662 placebo patients (5.6 %) (HR 0.08; 95 % CI 0.02–0.25). Major or clinically relevant non-major bleeding occurred in 36 dabigatran patients (5.3 %) and 12 placebo patients (1.8 %) (HR 2.92; 95 % CI 1.52–5.6) (Table 16). Acute coronary syndrome occurred in 1 patient in both groups [111].

### **Guidance Statement**

*In long term therapy of VTE, dabigatran was as effective as warfarin and superior to placebo in prevention of recurrent thromboembolism. There was a trend toward reduced major bleeding with dabigatran compared with warfarin but major bleeding was 3-fold higher with dabigatran than placebo. We suggest that these data establish dabigatran as a viable option to vitamin K antagonists for long term therapy of VTE.*

*Rivaroxaban* was investigated in long term treatment of VTE in the double-blind, double dummy EINSTEIN extension study. In this study which compared rivaroxaban 20 mg daily to placebo, 8 rivaroxaban recipients (1.3 %) and 42 placebo recipients (7.1 %) suffered recurrent VTE (HR 0.18; 95 % CI 0.09–0.39). Major bleeding occurred in 4 rivaroxaban patients (0.7 %) and no placebo recipients. Major or clinically relevant non-major bleeding occurred in 36 rivaroxaban patients (6.0 %) and 7 placebo patients (1.2 %) (HR 5.19; 95 % CI 2.3–11.7) (Table 16) [40].

### **Guidance Statement**

*In long term treatment of VTE (after 3–6 months of therapy), rivaroxaban was more effective than placebo but associated with 5 fold increase in major or clinically relevant non-major bleeding. We suggest that these data support the use of rivaroxaban in the long term treatment of VTE in candidates suitable for anticoagulation.*

*Apixaban*

In the AMPLIFY-EXT study, patients with VTE who had completed 6–12 months of therapy were randomized in a double blind fashion to either 2.5 or 5 mg of apixaban twice daily or placebo for 12 months [45]. Recurrent VTE occurred in 73 of 829 placebo recipients (8.8 %), 14 of 840 patients receiving 2.5 mg of apixaban (1.7 %; RR vs. placebo 0.19; 95 % CI 0.11–0.33) and 14 of 813 patients receiving 5 mg of apixaban twice daily (1.7 %; RR vs. placebo 0.20; 95 % CI 0.11–0.34). Non-VTE related cardiovascular death, myocardial infarction or stroke were similar between treatment groups. Major bleeding occurred in 4 patients on placebo (0.5 %), 2 patients on apixaban 2.5 mg twice daily (0.2 %; RR vs. placebo 0.49; 95 % CI 0.09–2.64) and 1 patient on apixaban 5 mg twice daily (0.1 %; RR vs. placebo 0.25; 95 % CI 0.03–2.24). Major or clinically relevant non-major bleeding occurred in 22 placebo recipients (2.7 %), 27 patients taking apixaban 2.5 mg twice daily (3.2 %; RR vs. placebo 1.20; 95 % CI 0.69–2.10) and 35 patients taking apixaban 5 mg twice daily (4.3 %; RR vs. placebo 1.62; 95 % CI 0.96–2.73) (Table 16) [45].

### **Guidance Statement**

*In long term treatment of VTE (after 6 months of therapy), apixaban was more effective than placebo and associated with a similar risk of major or clinically relevant non-major bleeding. We suggest that these data support the use of apixaban for the long term treatment of VTE in patients who are appropriate candidates. The option of a reduced dose for long term secondary prevention may be attractive for some patients.*

(d) *Aspirin* has been compared to placebo in 2 double-blind randomized controlled trials for prevention of VTE in patients with unprovoked VTE who were not considered candidates for long term anticoagulation therapy [123, 124]. The WARFASA study randomized 402 patients with a first episode of unprovoked VTE who had completed 6–18 months of oral anticoagulation to aspirin 100 mg daily or placebo. During a median treatment period of 24.6 months, recurrent VTE occurred in 28 of 205 aspirin recipients and 43 of 197 placebo recipients (6.6 % vs. 11.2 % per year; HR 0.58; 95 % CI 0.36–0.93). One patient in each group had major bleeding [123]. The ASPIRE study randomly assigned 822 patients with a first episode of unprovoked VTE to aspirin or placebo. During a median follow up duration of 37.2 months, 57 of 411 aspirin recipients and 73 of 411 placebo recipients suffered recurrent VTE (4.8 % per year vs. 6.5 % per year; HR 0.74; 95 % CI 0.52–1.05). Aspirin reduced the rate of the pre-specified composite outcome of VTE, MI, stroke and cardiovascular death by 34 % (5.2 % per year vs. 8.0 % per year, HR 0.66; 95 % CI 0.48–0.92). Major or clinically relevant non-major bleeding was similar (aspirin 1.1 % per year vs. placebo 0.6 % per year, p = 0.22) [124] (Table 16). An individual patient level data analysis of both trials found that aspirin therapy reduced recurrent VTE (5.1 % per year vs. 7.5 % per year; HR 0.68; 95 % CI 0.51–0.90, p = 0.008) including DVT (HR 0.66; 95 % CI 0.41–0.92, p = 0.01) and PE (HR 0.66; 95 % CI 0.41–1.06, p = 0.08). Major bleeding was low (aspirin 0.5 % per year vs. placebo 0.4 % per year). When adjusted for adherence to treatment, recurrent VTE was reduced by 42 % by aspirin therapy (HR 0.58; 95 % CI 0.40–0.85, p = 0.005). Similar relative risk reductions were seen in men and older patients [125]. These studies establish aspirin as an alternative treatment option for the long term treatment of patients with unprovoked VTE who have completed at least 3 months of anticoagulation. The risk of bleeding appears to be less than long term oral anticoagulation although antithrombotic efficacy is also substantially less. Therefore, aspirin may be a useful option for patients judged to be at higher risk for bleeding than recurrent VTE.

### **Guidance Statement**

*After an initial 6–18 months of anticoagulation, aspirin 100 mg daily was associated with a 34 % reduction in the relative risk of recurrent VTE (from 7.5 to 5.1 %) compared with placebo. Major bleeding was similar in both groups. Therefore, we suggest that aspirin should be considered an option for patients at risk for recurrent VTE who are not considered appropriate candidates for long term anticoagulation or who chose to discontinue anticoagulation.*

(11) What is the best treatment of patients who have recurrent VTE in spite of anticoagulation?

Choosing the best treatment for a patient who has suffered recurrent VTE requires confirmation that a recurrent event has indeed occurred, verifying that the patient was therapeutic at the time of recurrence and looking for the presence of clinical conditions associated with an increased risk for recurrent thromboembolism despite anticoagulation (Table 17). Symptoms (e.g. leg swelling, crampy pain, warmth, dyspnea and chest pain) concerning for VTE are common post thrombotic syndrome symptoms. Therefore objective documentation of recurrence thrombosis is essential to avoid unnecessarily discarding an effective treatment. For patients with a new DVT, documentation of an increase in thrombus burden (involvement of previously uninvolved vascular territories or an increase in thrombus diameter of 4 mm or more). In the event of an increase in thrombus diameter of 1–3.9 mm, repeat imaging in 1 week or venography is warranted [126]. Recurrent PE is diagnosed if CT angiography documents a new filling defect in a segmental or larger artery or new segmental mismatched defect is identified on ventilation perfusion scanning [127].

**Table 17:** Reasons for recurrent VTE despite anticoagulation

Anatomic compression (i.e. May-Thurner syndrome, Thoracic Outlet syndrome, etc.)
Antiphospholipid syndrome
Cancer
Dysfibrinogenemia
Heparin-induced thrombocytopenia
Myeloproliferative Neoplasm-uncontrolled (e.g. Polycythemia Vera, Essential Thrombocythemia)
Paroxysmal Nocturnal Hemoglobinuria
Subtherapeutic anticoagulation

If a new event is confirmed it is important to determine whether subtherapeutic anticoagulation was a contributing factor. For patients on a VKA, review of the recent INR values is essential. If subtherapeutic values are identified redoubled efforts to maintain therapeutic INR values are appropriate or perhaps a slight adjustment of the INR range upward (increase from INR 2–3 to 2.5–3.5) to reduce the chances of subtherapeutic INR values in the future. In patients on a VKA it is also important to determine whether they have one of a selected group of thrombophilic disorders that predisposes to recurrent VTE despite therapeutic anticoagulation. Considerations include cancer, antiphospholipid syndrome, dysfibrinogenemia and delayed heparin induced thrombocytopenia as well as anatomic reasons for recurrent DVT. If antiphospholipid syndrome or dysfibrinogenemia is present, then the validity of the INR in the patient should be confirmed using a test that is not influenced by the coagulopathy, such as the chromogenic factor X activity assay. For patients with cancer, therapeutic dose LMWH should be used for therapy. For patients with delayed HIT, use of a direct thrombin inhibitor or fondaparinux with later transition to warfarin following HIT resolution should be considered. Consideration of surgical correction of anatomic vascular compression is warranted.

For patients on a VKA who suffer recurrent VTE without an identifiable cause, there are limited data for guidance but potential options include use of a higher INR target range (INR 2.5–3.5 or 3–4 depending upon the INR at the time of the thrombotic event) or switching to a LMWH or fondaparinux [119, 128–130]. Since DOACs were demonstrated to be equivalent to VKA in the treatment of VTE, additional data are needed to support their use in the setting of recurrent VTE during anticoagulation with VKA. For patients on LMWH, providers should make sure they have been taking the appropriate dose and refilling their prescriptions at appropriate intervals. LMWH (anti-Xa) levels are not usually practical as they are rarely obtained in a timely manner when the results would be useful. If patients are taking the appropriate dose then increasing the dose by 25 % is a common strategy used in cancer patients [131]. In patients taking enoxaparin, one can switch to 1 mg/kg twice daily dosing if 1.5 mg/kg once daily dosing had been used. Alternatively, one could switch to fondaparinux which has a longer half-life than LMWH. Rarely, cancer patients with Trousseau’s syndrome are not responsive to LMWH or fondaparinux in therapeutic doses. In these instances one might use IV UFH to assess the dose necessary for control of the coagulopathy and then transition to subcutaneous UFH adjusted to aPTT levels. Whenever recurrence occurs it is important to identify risk factors for thrombosis and then eliminate any removable risk factors (i.e. vascular compression causing stasis) [59]. For patients on a DOAC who experience recurrent VTE, providers should ask patients about missed doses prior to the event. Since DOACs have a short half-life, measuring anti-Xa levels (factor Xa inhibitors) or anti-IIa assays (Hemoclot^®^ thrombin inhibitor assay or ecarin clotting time) is not a practical strategy to assess adherence. If there are no identifiable reasons (i.e. cancer, anatomic vascular compression) for recurrence in a patient taking a DOAC for VTE, switching the patient to a VKA where adherence can be monitored may be preferable.

### **Guidance Statement**

*In patients with recurrent VTE despite anticoagulation, we suggest that it is important for providers to assess adherence to therapy and identify clinical conditions associated with anticoagulation failure including cancer, antiphospholipid syndrome, heparin-induced thrombocytopenia and vascular compression syndromes (May-Thurner syndrome, thoracic outlet syndrome). We suggest that higher-intensity anticoagulation (VKA INR 2.5–3.5 or 3–4 or based upon chromogenic factor X activity or escalated dose [125 % dose] LMWH), alternative forms of parenteral anticoagulation and therapies directed at restoring adequate blood flow are effective strategies to consider.*

(12) How can you assess the risk of recurrent VTE and anticoagulant-associated bleeding?

*Recurrent VTE* Risk factors for recurrent VTE include unprovoked VTE, certain hereditary thrombophilia, antiphospholipid syndrome, cancer, male gender, elevated D dimer on and off anticoagulation and the presence of residual venous thrombosis on duplex ultrasound [132]. As outlined above in the duration of therapy section of this chapter, the most important risk factor for recurrence is the presence or absence of situational prothrombotic triggers at the time of the VTE. Patients with unprovoked VTE are intrinsically hypercoagulable and remain at elevated risk for recurrent VTE if anticoagulation is discontinued. In contrast, patients who suffer VTE in the presence of potent situational triggers such as major surgery or trauma are at low risk of recurrent VTE [81]. If a patient still has active risk factors for VTE at the conclusion of therapy (relative immobility, persistent infections or hospitalization associated with surgical complications) then prolongation of the course of anticoagulation should be considered on a case-by-case basis. Patients with non-surgical risk factors for VTE are at intermediate risk for recurrence (4.2 % per year) [81]. In these patients, continuation of therapy is appropriate if the identifiable risk factors (active inflammatory bowel disease, etc.) are still present [132].

A prime example of this patient subgroup are cancer patients. Cancer patients with VTE are 3-fold higher risk of recurrence than patients without cancer [133]. Therefore, cancer patients with VTE should remain on anticoagulation until they are in remission and no longer on cancer therapy. Not surprisingly, the risk of recurrence varies with the type and extent of cancer. Patients with stage 1 or 2 cancer have a similar risk of recurrence as patients without cancer while patients with stage 3 or 4 disease are at significantly higher risk of recurrence. Patients with lung, gastrointestinal and genitourinary cancers were at greater risk for recurrence than other cancer sites [133]. Chee et al. also noted cancer stage and primary site were risk factors for recurrence in their population-based cohort study [134]. The understanding that not all cancer patients are at equal risk of recurrence has spurred the development of risk assessment models to identify patients at greater risk of recurrent VTE such as the Ottawa Risk Prediction Model [135]. More detailed discussion of treatment of VTE in cancer patients can be found in accompanying paper by Khorana et al.

As described above and the accompanying paper by Stevens et al., inherited thrombophilic disorders are associated with a risk of recurrent VTE which varies by the genetic defect. Hormonal therapy and pregnancy as well as the post-partum period represent risk factors for initial and recurrent VTE (see duration of therapy section above and the accompanying paper by Stevens et al.

Obesity has been associated with a 1.6 fold increased risk of recurrent VTE [136]. Therefore, weight loss should be included in VTE risk modification strategies for patients with VTE.

Residual vein obstruction (RVO) refers to the presence of residual thrombus at the site of an initial DVT after a defined period of anticoagulation. In a patient-level meta-analysis, RVO was associated with a 1.3 fold increased risk of recurrent VTE (95 % CI 1.06–1.65) among patients with unprovoked DVT. RVO measured at 3 months was associated with a higher risk of recurrent VTE (HR 2.17; 95 % CI 1.11–4.25) while RVO detected beyond six months was not a significant predictor of recurrence risk (HR 1.19; 95 % CI 0.87–1.61) [137]. Aside from its utility as a prognostic tool, duplex ultrasound can be used to establish a baseline at the end of therapy in case a patient reports symptoms potentially attributable to a new DVT [126, 127]. Since the symptoms of DVT and post-thrombotic syndrome can be difficult to differentiate, we obtain baseline duplex studies at the end of therapy in patients at high risk for recurrence who are discontinuing therapy [138].

An abnormal D dimer is associated with a 2.6 fold (95 % CI 1.90–3.52) increased risk of recurrent VTE among patients with unprovoked events regardless of age or cut point (500 vs. 250 µg/L) [139]. In a prospective management study of patients with unprovoked VTE who discontinued anticoagulation, Cosmi et al. found that patients with persistently abnormal D dimer studies at and beyond 90 days post-discontinuation of AC had a 9 fold higher risk of recurrence than patients with normal D dimers (Recurrent VTE 27 per 100 patient years [95 % CI 12–48] vs. 2.9 per 100 patient years [95 % CI 1–7]; HR 9.38, p = 0.0004) [140]. Palareti et al. also found that D dimer testing identified patients with an initial unprovoked VTE or one associated with minimal risk factors who were at risk for recurrent VTE (8.8 % per patient-year vs. 3.0 % per patient-year; HR 2.92; 95 % CI 1.87–9.72; P = 0.0006) [141]. Using serial qualitative D dimer testing in patients with an unprovoked VTE or one provoked by estrogen therapy, Kearon and colleagues noted that women with a negative D dimer who had an estrogen associated VTE were at low risk for recurrence (0 %,95 % CI 0.0–3.0 %). Men (Recurrent VTE 9.7 % per patient year; 95 % CI 6.7–13.7 %) and women with unprovoked VTE (5.4 % per patient year; 95 % CI 2.5–10.2 %) remained at substantial risk of recurrence despite two negative D dimers [98]. In patients with provoked VTE, Cosmi et al. found that an abnormal D dimer at the time of discontinuation of AC (Day 0) (11.1 VTE per 100 patient-years [95 % CI 4–24] vs. 2.2 VTE per 100 pt.-years [95 % CI 1–4]; adj. HR 4.2 [1.2–14.2]) and 30 days after discontinuation of AC (Day 30) (6.7 VTE per 100 patient-years [95 % CI 3–12] vs. 1.5 VTE per 100 pt.-years [95 % CI 0–3]; adj. HR 3.8[1.2–12.1]) were both associated with an increased risk of recurrence [142].

Other global measures of activated coagulation also have been associated with recurrence risk. In a prospective study of 914 patients with spontaneous VTE, Hron et al. found that thrombin generation (TG) less than 300 nM (nM) (RR 0.37 [95 % CI 0.20–0.67]) and 300–400 nM (RR 0.45 [95 % CI 0.28–0.73] were associated with a reduced risk of recurrent VTE compared to patients with TG greater than 400 nM [143]. In a prospective study of 188 patients with unprovoked VTE or VTE associated with a non-surgical trigger, Besser et al. also noted that endogenous thrombin potential (ETP) greater than the 50^th^ percentile was associated with an almost 3 fold greater risk of recurrence (HR 2.9, 95 % CI 1.3–6.6, cumulative recurrence at 4 years 27 vs. 11 %). A high ETP remained a significant predictor of recurrent VTE even after adjustment for D dimer level, presence of thrombophilia, sex and whether or not the first event was unprovoked (HR 2.6, 95 % CI 1.2–6.0) [144]. Tripodi et al. found that patients with unprovoked VTE who had an ETP of greater than 960 nM times minutes or peak TG greater than 193 nM measured in the presence of thrombomodulin were at increased risk for recurrent VTE (Hazard ratios (HR) of 3.41 (95 % CI 1.34–8.68) and 4.57 (95 % CI 1.70–12.2), respectively) [145]. Patients with unprovoked VTE who had an aPTT ratio ≥0.95 measured a median of 13 months after discontinuation of anticoagulation have been noted to have a lower risk for recurrent VTE of 0.58 (95 % CI 0.39–0.87, P = 0.009) after adjustment for sex, age, FVL and PGM compared to patients with an APTT ratio <0.95 [146]. Among 544 patients with unprovoked VTE and P selectin levels above the 75th percentile, the probability of recurrence after 4 years of follow up was 20.6 % (95 % CI 12.6–28.5) versus 10.8 % (95 % CI 7.2–14.3) among patients with lower values for an adjusted risk of recurrence of 1.7-fold (95 % CI 1.0–2.9, p = 0.045) [147].

Ideal risk assessment models for the risk of recurrent VTE should be derived from prospective observational studies, incorporate both clinical and laboratory risk factors that are available in a wide variety of practice settings and be validated prospectively in different patient populations. Several risk models for the determination of the clinical risk of recurrent VTE in patients with unprovoked VTE have been developed. Marc Rodger and colleagues examined multiple clinical and laboratory risk factors for recurrent VTE in 646 consecutive patients with a first episode of unprovoked VTE. In their analysis, signs of post-thrombotic syndrome (redness, hyperpigmentation or edema of the leg), age ≥ 65 years, a body mass index ≥30 kg/M2, and a D dimer ≥250 µg/L were identified as risk factors for recurrence. Women with fewer than 2 of these risk factors were at low risk for recurrence (1.6 % per year; 95 % CI 0.3–4.6 %) while those with 2 or more had an annual recurrence risk of 14.1 % per year (95 % CI 10.9–17.3 %). They were unable to identify a low risk group of men in their analysis. They have dubbed their clinical prediction model “Men Continue and HERDOO2” [148].

Eichinger and colleagues conducted a multicenter prospective cohort study of 929 consecutive patients with a first episode of unprovoked VTE to develop a risk model to identify patients at risk for recurrence. Risk factors for recurrence included male sex (hazard ratio 1.90, 95 % confidence interval 1.31–2.75), proximal deep vein thrombosis (hazard ratio vs. distal 2.08, 95 % confidence interval 1.16–3.74), pulmonary embolism (hazard ratio vs. distal thrombosis 2.60, 95 % confidence interval 1.49–4.53), and elevated levels of D-dimer (hazard ratio per doubling 1.27, 95 % confidence interval 1.08–1.51). Using these factors they have developed a web-based risk prediction calculator (Vienna Prediction Model for Recurrent VTE) that is available on the web (http://cemsiis.meduniwien.ac.at/en/kb/science-research/software/clinical-software/recurrent-vte/) [149].

In 2012 Tosetto and colleagues published the DASH prediction score based upon a patient level meta-analysis of 1818 patients with unprovoked VTE who participated in 7 prospective studies. DASH is an acronym that stands for each of the elements of the prediction rule-abnormal D dimer post-anticoagulation (2 points), age ≤ 50 years (1 point), male sex (1 point) and hormone use at the time of the initial VTE in women (−2 points). Annual recurrence rates associated with DASH scores of −2 to 4 ranged from 1.8 to 19.9 % per year [97]. External validation of each of these models in a variety of patient populations in prospective studies is necessary. Rodger and colleagues and Eichinger et al. are currently conducting prospective validation studies of their models.

### **Guidance Statement**

*We suggest that patients with unprovoked VTE should be considered intrinsically thrombophilic and long term anticoagulation should be considered. When assessing the risk of recurrent VTE in patients with provoked VTE, it is important to determine whether provoking factors persist. If such factors are still present, we suggest that continued anticoagulation should be considered if bleeding risk is not excessive.*

We suggest that D dimer represents a promising global measure of pro-thrombotic potential that can be used to risk stratify patients for their future risk of VTE. However, we believe it is important to recognize that different D dimer assays may have different performance characteristics in regards to VTE risk assessment. In addition, the impact of age on the D dimer results and VTE risk prediction remains incompletely characterized. The value of other global laboratory measures (endogenous thrombin potential) and imaging studies remains to be established. Multimodality risk assessment models appear to be an effective approach to risk stratification of patients with unprovoked VTE. Further validation of these risk assessment tools is underway.

*Anticoagulation-associated bleeding*

In determining the appropriate duration of anticoagulant therapy for VTE it is important to assess not only the risk of recurrent VTE but also the risk of bleeding associated with continued anticoagulation. Several different models have been developed primarily by studying patients with atrial fibrillation to determine the risk of bleeding associated with anticoagulation with vitamin K antagonists (HEMORR_2_HAGES, HAS-BLED, ATRIA). However the clinical characteristics of patients with atrial fibrillation and VTE differ. Therefore, these bleeding risk prediction models may not be as predictive in patients with VTE. Several bleeding risk assessment models have been developed specifically for VTE patients. However, the performance of these models in assessing bleeding risk has been modest [150]. In addition, none of these models has been used or validated in conjunction with DOACs. Therefore, at this point, it is premature to recommend the use of bleeding risk assessment models to determine the duration of anticoagulation for patients with VTE. Development of new bleeding risk prediction models as well as composite tools that identify patients at increased risk for multiple adverse events (e.g., thrombosis, bleeding) remain an important area of investigation.

### **Guidance Statement**

*While a bleeding risk assessment is important to the decision on the duration of anticoagulation, we suggest that it is premature to use formal bleeding risk assessment models to identify patients who should discontinue anticoagulation. Development of better risk prediction models remains a priority.*

*Balancing the risks and benefits* Treatment of VTE is associated with benefits (reduction in recurrent VTE and its morbidity and mortality) as well as risks (bleeding complications, negative economic and life style impact). Therefore, decision-making on the duration of anticoagulation must be made jointly with the patient taking into account their beliefs and preferences. For this conversation to be productive and informed the patient must be well educated by the provider about venous thromboembolism and its treatment so that he/she can make an informed decision. A variety of excellent resources are available to improve patient understanding of VTE including the National Blood Clot Alliance, Clot Connect, Thrombosis Canada and the Centers for Disease Control and Prevention just to name a few. In the clinic, we review the pathophysiology of VTE with patients, the presenting signs and symptoms and the individual risk factors for VTE and the risk factors and signs and symptoms of anticoagulation associated bleeding so that patients can make an informed decision and know when it is appropriate to contact healthcare providers. For patients with unprovoked VTE we generally recommend long term anticoagulation unless they are non-adherent or have had bleeding complications. If the patient favors discontinuation of anticoagulation, we use the Vienna Prediction Model or data derived from the literature to determine an estimate of the patients risk for recurrent thromboembolism so that they have a rough idea of their risk of recurrence off anticoagulation [96, 149, 151]. For patients who suffered VTE in the setting of a major transient risk factor (major surgery or trauma) we generally discontinue anticoagulation after 3–6 months of therapy. For patients with non-surgical triggered VTE, we treat for at least 3–6 months or longer if the risk factors for their event remain unresolved. Table 18 summarizes these guidance statements.

**Table 18:** Summary of guidance statements

Question	Guidance statement
How is the diagnosis of deep vein thrombosis and pulmonary embolism established?	We suggest the use of validated pre-test probability models in conjunction with D dimer testing and selective use of objective diagnostic imaging to increase the cost-efficiency and accuracy of VTE diagnosis
Which patients require hospitalization versus initial outpatient therapy for the management of VTE?	We suggest that most patients with DVT and many patients with PE can be managed as outpatients. PE patients should be risk stratified to determine appropriate management. A variety of laboratory tests and imaging modalities as well as clinical risk prediction models are available to identify PE patients who are suitable for outpatient management. Further research is needed to identify the optimal approach to risk stratification of PE patients
What are the therapeutic options for the acute treatment of venous thromboembolism?	With the variety of treatment options available, we recommend that the acute therapy of VTE should be customized to suit the unique clinical circumstances of the individual patient. We suggest that unfractionated heparin may be preferable for inpatients with planned invasive procedures, recent major bleeding episodes or impaired renal function as well as underweight and morbidly obese patients although several members of panel felt there were insufficient data to support this suggestion. LMWHs are convenient options for inpatient and outpatient therapy. DOACs are optimized for outpatient therapy of VTE We suggest that systemic and catheter-directed pharmacomechanical thrombolytic therapy are effective options for treatment of acute extensive proximal DVT and massive PE that can rapidly reduce thrombus burden. Given the greater risks of bleeding associated with these approaches, we recommend that a careful assessment of the risks and benefits of therapy should be performed in each patient prior to the initiation of thrombolytic therapy
Which patients are candidates for a DOAC?	Dabigatran When used after a 5–10 day initial course of parenteral anticoagulation, dabigatran is as effective as warfarin in the acute and short term treatment of VTE. We suggest dabigatran as an alternative to vitamin K antagonists for the short term therapy of VTE. In some studies, dabigatran has been associated with an increased risk of acute coronary syndrome and gastrointestinal bleeding compared with vitamin K antogonists Rivaroxaban Rivaroxaban is as effective as LMWH/VKA in the treatment of DVT and PE. We suggest rivaroxaban as an alternative to LMWH/VKA for the acute and short term treatment of VTE in appropriate patients. No increase in acute coronary syndrome or gastrointestinal bleeding has been seen with rivaroxaban, however GI bleeding may be more common in patients age 75 and older Apixaban Apixaban is as effective as LMWH/VKA in the treatment of DVT and PE and associated with less major bleeding and major or clinically relevant non-major bleeding. We suggest apixaban as an alternative to LMWH/VKA in the acute and short term treatment of VTE in appropriately selected patients. No increase in acute coronary syndrome or gastrointestinal bleeding has been seen with apixaban Edoxaban After an initial 5–10 days of LMWH or UFH, edoxaban is as effective as LMWH/VKA in the treatment of acute DVT and PE but associated with less major or clinically relevant non-major bleeding. We suggest edoxaban as an alternative to VKA for the short-term treatment of VTE in appropriately selected candidates
What is the role of vena cava filters if the patient is not a candidate for anticoagulation?	We suggest that vena cava filters should be considered in any patient with acute VTE (within 4 weeks) who cannot be treated with anticoagulation. We suggest that retrievable filters are strongly preferred as most patients have only temporary contraindications to anticoagulation. Filters should be retrieved once anticoagulation can be reinitiated preferably within 6 months of placement. Patients with filters should be closely monitored in a structured program to facilitate retrieval and minimize the number of patients lost to follow up Following anticoagulation-associated gastrointestinal bleeding, we suggest that anticoagulation can be re-initiated as early as 7 days after cessation of bleeding and treatment of causal lesions. Following anticoagulation associated ICH, we suggest resumption of anticoagulation no sooner than 10 weeks post-bleed. Further investigation of this topic is warranted
How is upper extremity VTE treated?	Identification and elimination of trigger factors when feasible is important to reduce the incidence of recurrent upper extremity DVT. For CVC-associated DVT, we suggest that anticoagulation without CVC removal is the treatment of choice. If symptoms fail to resolve, CVC removal can be considered. We suggest that anticoagulation should be continued for at least 3 months or the duration of the CVC whichever is longer. At least 3 months of anticoagulation is appropriate for pacemaker wire-associated VTE The committee was divided as to the optimal approach to treatment of TOS/PSS-associated upper extremity DVT. The benefits of rib resection/scalenectomy following thrombolysis and anticoagulation remain to be rigorously demonstrated. Therefore, providers should consider therapy for TOS/PSS on a case-by-case basis until higher quality data are available. We suggest that TOS/PSS-associated upper extremity DVT warrants anticoagulation for at least 3 months. Treatment of upper extremity DVT associated with extrinsic compression due to cancer or infection should include treatment of the underlying disease in addition to anticoagulation
When is ambulation/exercise safe after DVT/PE?	We suggest that ambulation is safe in patients with acute DVT ± PE after initiation of anticoagulation
Is the use of graduated compression stockings safe after acute DVT/PE?	We suggest that GCS do not increase the risk of recurrent thromboembolism in patients with acute VTE. We suggest that GCS do not have any beneficial effect on leg discomfort associated with acute DVT
What is the recommended duration of therapy for a patient with distal DVT?	We suggest treatment of distal DVT with anticoagulation versus observation. We suggest a duration of therapy of 3 months. In patients with contraindications to anticoagulation, we favour repeat duplex surveillance in 1 week rather than vena cava filter insertion
What is the recommended duration of therapy for a patient with a surgically provoked VTE?	We suggest that 3 months of anticoagulation is adequate for surgical risk factor-associated VTE unless risk factors for recurrence persist
What is the recommended duration of therapy for a pregnancy or estrogen-associated VTE?	We suggest that patients with pregnancy-associated VTE should be treated for the duration of the pregnancy and the post-partum period (up to 12 weeks post-partum) or as long as dictated by the VTE, whichever is longer. Patients with pregnancy-associated VTE are at high risk for recurrent VTE with subsequent pregnancies, therefore we suggest that thromboprophylaxis for the duration of the pregnancy and post-partum period should be strongly considered Patients with hormone-associated VTE appear to be at lower risk for recurrent VTE particularly if their D dimer is negative at the end of therapy and 1 month after discontinuing anticoagulation. Therefore, we suggest that long term anticoagulation beyond 3–6 months may not be associated with a favorable risk: benefit balance if hormonal therapy has been discontinued. If hormonal therapy is medically necessary, we suggest that anticoagulation should be continued as these patients are at high risk for recurrent VTE
What is the recommended duration of therapy for a medical illness-associated VTE?	We suggest that patients with medical-illness associated VTE should be treated for at least 3 months or as long as the medical risk factors for VTE remain present
What is the recommended duration of therapy for a travel-associated VTE?	Travel (>4 h duration) is a modest and transient risk factor for VTE. Therefore, VTE should only be ascribed to air travel if it presents within 4 weeks of travel and is not associated with other concomitant triggers. In the absence of other precipitants, we suggest that travel-associated VTE should be treated for at least 3 months. We suggest that travel thromboprophylaxis be considered for future travel in these patients
What is the recommended duration of therapy for a malignancy-associated VTE?	Active cancer is a potent risk factor for VTE that varies with the type and extent of cancer and its treatment. Therefore, we suggest anticoagulation be continued as long as the underlying cancer is active or under treatment
What is the recommended duration of therapy for a patient with unprovoked VTE?	Patients with unprovoked VTE are at high risk for recurrence so we suggest long term anticoagulation. As there is limited information on the risks and benefits of anticoagulation beyond 2 years, we suggest that providers reassess patients on long term anticoagulation on an annual basis
What are the therapeutic options for long term treatment of DVT/PE?	Vitamin K antagonists Adjusted dose vitamin K antagonists (INR 2–3) reduce the relative risk of recurrent VTE by 88 %, but they are associated with 2.6 fold increase in major bleeding compared with placebo. Consequently, it is important to assess the risks and benefits of long term anticoagulation on a case-by-case basis. Since low intensity (INR 1.5–2) anticoagulation is associated with a similar risk of major bleeding, we prefer standard intensity anticoagulation for long term therapy of VTE LMWH/Fondaparinux Evidence indicates that LMWH is as effective as VKA in the reduction of recurrent VTE but associated with a reduced risk of major bleeding. Limited experience with fondaparinux in the long term treatment of VTE suggests that it is as effective as LMWH in the prevention of recurrent VTE. Fondaparinux may cause more bleeding than VKA in patients without cancer. We suggest that LMWH and fondaparinux are acceptable alternatives to VKA for treatment of VTE Direct Oral Anticoagulants Dabigatran In long term therapy of VTE, dabigatran was as effective as warfarin and superior to placebo in prevention of recurrent thromboembolism. There was a trend toward reduced major bleeding with dabigatran compared with warfarin but major bleeding was 3-fold higher with dabigatran than placebo. We suggest that these data establish dabigatran as a viable option to vitamin K antagonists for long term therapy of VTE Rivaroxaban In long term treatment of VTE (after 3–6 months of therapy), rivaroxaban was more effective than placebo but associated with 5 fold increase in major or clinically relevant non-major bleeding. We suggest that these data support the use of rivaroxaban in the long term treatment of VTE in candidates suitable for anticoagulation Apixaban In long term treatment of VTE (after 6 months of therapy), apixaban was more effective than placebo and associated with a similar risk of major or clinically relevant non-major bleeding. We suggest that these data support the use of apixaban for the long term treatment of VTE in patients who are appropriate candidates. The option of a reduced dose for long term secondary prevention may be attractive for some patients Aspirin After an initial 6–18 months of anticoagulation, aspirin 100 mg daily was associated with a 34 % reduction in the relative risk of recurrent VTE (from 7.5 to 5.1 %) compared with placebo. Major bleeding was similar in both groups. Therefore, we suggest that aspirin should be considered an option for patients at risk for recurrent VTE who are not considered appropriate candidates for long term anticoagulation or who chose to discontinue anticoagulation
What is the best treatment of patients who have recurrent VTE in spite of anticoagulation?	In patients with recurrent VTE despite anticoagulation, we suggest that it is important for providers to assess adherence to therapy and identify clinical conditions associated with anticoagulation failure including cancer, antiphospholipid syndrome, heparin-induced thrombocytopenia and vascular compression syndromes (May-Thurner syndrome, thoracic outlet syndrome). We suggest that higher-intensity anticoagulation (VKA INR 2.5-3.5 or 3–4 or based upon chromogenic factor X activity or escalated dose [125 % dose] LMWH), alternative forms of parenteral anticoagulation and therapies directed at restoring adequate blood flow are effective strategies to consider
How can you assess the risk of recurrent VTE and anticoagulant-associated bleeding?	We suggest that patients with unprovoked VTE should be considered intrinsically thrombophilic and long term anticoagulation should be considered. When assessing the risk of recurrent VTE in patients with provoked VTE, it is important to determine whether provoking factors persist. If such factors are still present, we suggest that continued anticoagulation should be considered if bleeding risk is not excessive We suggest that D dimer testing represents a promising global measure of pro-thrombotic potential that can be used to risk stratify patients for their future risk of VTE. However, we believe it is important to recognize that different D dimer assays may have different performance characteristics in regards to VTE risk assessment. In addition, the impact of age on the D dimer results and VTE risk prediction remains incompletely characterized. The value of other global laboratory measures (endogenous thrombin potential) and imaging studies remains to be established. Multimodality risk assessment models appear to be effective approach to risk stratification of patients with unprovoked VTE. Further validation of these risk assessment tools is underway Anticoagulation-associated bleeding While a bleeding risk assessment is important to the decision on the duration of anticoagulation, we suggest that it is premature to use formal bleeding risk assessment models to identify patients who should discontinue anticoagulation. Development of better risk prediction models remains a priority
